# Laser Processing of Fe-Cr-B Alloys: Microstructure Evolution, Non-Equilibrium Solidification and Wear–Corrosion Performance

**DOI:** 10.3390/ma19132767

**Published:** 2026-06-30

**Authors:** Lei He, Changle Zhang, Jiang Ju, Zhizu Zhang, Jintao Liu, Huajun Zhang

**Affiliations:** 1 School of Materials Science and Engineering, Shanghai Jiao Tong University, Shanghai 200240, China; helei18336759694@163.com (L.H.); jujiang_1990@163.com (J.J.); 2 Luoyang Ship Material Research Institute, Luoyang 471023, China; liujintao725@163.com; 3 National Key Laboratory of Marine Corrosion and Protection, Luoyang 471023, China; 4 Sodium-Ion Battery Energy Storage Technology Key Laboratory Cultivation Base Jointly Constructed by the Department and City of Shanxi Province, Yangquan 045000, China; zhangzhizu6688@163.com

**Keywords:** Fe-Cr-B alloy, laser additive manufacturing (LAM), laser powder bed fusion (LPBF), directed energy deposition (DED), laser cladding, casting, wear resistance, corrosion resistance, microstructure control

## Abstract

**Highlights:**

**What are the main findings?**

- Conclusions supported by direct bulk LAM (LPBF/DED) experimental evidence:
- Bulk laser additive manufacturing suppresses elemental segregation, reduces internal defects, and optimizes the balance between the hardness and toughness of Fe-Cr-B alloys.
- Optimized LPBF/DED processing enables Fe-Cr-B alloys to achieve relative density over 98% and remarkable boride refinement.
- Conclusions inferred indirectly from laser cladding literature:
- Extreme non-equilibrium rapid solidification refines boride phases and restrains continuous network segregation in Fe-Cr-B alloys.
- Laser-induced microstructure modification alleviates the intrinsic brittleness originating from coarse network borides in conventionally cast alloys.
- Microstructure regulation via composition design, process optimization and post-treatment can synergistically enhance wear and corrosion resistance.

**What are the implications of the main findings?**

- Laser processing opens a new paradigm for fabricating high-performance Fe-Cr-B alloy components via both surface coating and bulk near-net shaping.
- The proposed research roadmap facilitates the industrialization and commercialization of LAM/laser cladding Fe-Cr-B alloys.
- It promotes the transformation of wear-resistant materials from conventional macro-forming toward precise microstructural manufacturing.

**Abstract:**

Fe-Cr-B alloys are recognized as candidate wear- and corrosion-resistant materials strengthened by high-hardness boride phases. Conventional casting produces coarse continuous network borides and severe elemental segregation under near-equilibrium slow solidification (10^−1^–10^2^ K/s), resulting in high brittleness and limited service reliability. Laser processing includes laser cladding (10^3^–10^6^ K/s), LPBF/DED (10^6^–10^8^ K/s) and laser remelting, which feature extreme non-equilibrium rapid solidification but differ significantly in thermal gradient G, solidification rate R, and phase evolution behavior. To avoid over-extrapolation, this review strictly classifies evidence into direct LPBF evidence, direct DED evidence, laser cladding evidence, casting evidence, and indirect inference. Quantitative comparisons reveal that laser cladding refines borides from 150 to 300 μm to 10.8–20 μm, while DED further achieves 1–5 μm equiaxed grains and relative density > 98%. Meanwhile, laser-cladding Fe-Cr-B coatings achieve a maximum hardness of ~1052 HV_0.5_, and ~18% higher wear resistance and ~70% lower cavitation mass loss compared with cast counterparts. Non-equilibrium mechanisms including solute trapping, interface absolute stability, constitutional undercooling, and columnar-to-equiaxed transition (CET) controlled by the G^n^/R ratio are systematically analyzed. Thermal–solutal coupling, grain nucleation, and boride precipitation kinetics under rapid cooling are emphasized. Current limitations include incomplete non-equilibrium thermodynamic databases, insufficient standardization, limited post-processing (heat treatment, HIP) studies, and missing unified performance datasets. Future directions are proposed toward quantitative phase-field modeling, standardized tribocorrosion characterization, high-throughput experiments, and machine learning-assisted optimization. This review provides a rigorous analytical framework for the composition–process–microstructure–performance design of laser-processed Fe-Cr-B alloys.

## 1. Introduction

In the fields of high-end equipment manufacturing and major engineering projects, the demand for wear-resistant components with long service life and high reliability has become a national strategic priority. Wear and corrosion are the two dominant causes of material failure, acting as costly invisible killers in industrial systems worldwide, with associated economic losses remaining excessively substantial. Under extreme service conditions such as deep-sea drilling, heavy-duty mining machinery operation, and aero-engine operation, components endure the synergistic coupling effects of wear, corrosion, impact, and high stress [1,2]. The performance of traditional wear-resistant materials (e.g., high-chromium cast iron) has neared their theoretical limits, which makes it difficult to meet the exponentially growing demands of modern equipment for higher reliability and longer service life. This severe challenge is driving a profound paradigm shift from the empirical modification of existing materials to the development of next-generation materials based on microstructural design and precise control.

Driven by the ever-increasing demands and technological advances, wear-resistant materials have evolved from austenitic manganese steel and high-chromium (Cr) cast iron to a new and widely investigated category: boron (B)-alloyed steels with improved corrosion resistance [3,4,5]. (Casting). Compared with high-Cr cast iron and stainless steel, Fe-Cr-B alloys have achieved a significant technological breakthrough. Their unique advantage lies in the use of boride phases as the primary hard phases instead of carbides, thus achieving an excellent balance of wear resistance, corrosion resistance, and moderate impact toughness. This performance synergy mainly depends on the type, morphology, and distribution of boride phases. For example, studies by research groups such as Hanguang Fu [3] focused on the role of Cr/B ratio on boride formation, while Wenjiang Ding [4] investigated the corrosion and wear resistance performance under elevated temperatures. At present, Fe-Cr-B alloys are widely used in the fabrication of various wear- and corrosion-resistant components, including glass molds, ball mill liners, guide rolls, hammers, grinding balls, and high-boron high-speed steel rolls [5,6,7,8,9,10,11]. However, the fundamental limitation of traditional casting processes for fabricating Fe-Cr-B alloys is the low solidification rate, which results in the formation of coarse boride phases with a continuous network distribution, as well as severe compositional segregation and a high hot cracking tendency. The inherent trade-off between boride phase strengthening and network-induced embrittlement has become a key bottleneck limiting the performance improvement of these alloys [12,13,14,15,16,17,18]. (Casting). Laser additive manufacturing (LAM) presents a promising solution to these challenges.

In recent decades, laser processing technology has attracted widespread attention due to customized forming capability and flexible microstructure regulation [19]. (Indirect inference) In this review, laser processing is divided into three independent but correlated technical types:

(1) Bulk laser additive manufacturing: mainly including LPBF and DED, used for near-net shaping of structural bulk parts;
(2) Laser cladding: surface modification technology, regarded as the technical precursor of bulk LAM;
(3) Laser remelting: secondary surface strengthening technology, often combined with cladding or LAM to optimize interface and microstructure. (Laser cladding/Indirect inference).

All the above technologies rely on high-energy laser beam irradiation and rapid solidification, but there are obvious differences in cooling rate, thermal cycle characteristics, defect types and mechanical anisotropy, and research conclusions cannot be arbitrarily generalized across different processes. Furthermore, laser processing exhibits notable advantages including a short manufacturing cycle, high precision, good repeatability, and reduced susceptibility to the thermal gradients and process-induced residual stresses inherent to laser or electron beam processing [1,20,21]. Additionally, it has been demonstrated that LAM can control the magnitude and stability of the friction coefficient over a wide load range [7,22]. (Indirect inference).

Compared with conventional casting, laser processing is characterized by high-energy beam action, extreme non-equilibrium rapid solidification and layer-by-layer manufacturing. (Indirect inference). Among them, laser cladding can significantly refine the matrix and boride phases of Fe-Cr-B coatings, inhibit Cr segregation and improve microstructure uniformity and service performance. Relevant microstructure evolution laws are mostly summarized from laser cladding results [20,21]. (Laser cladding). By contrast, research on the direct DED bulk fabrication of Fe-Cr-B alloys is still in the initial stage, but existing direct experimental evidence has verified its microstructure refinement potential [20,21,22]. (DED). The corrosion mechanism transforms from selective corrosion to pitting corrosion, and improving passive film stability is the key to enhancing corrosion resistance [12,17]. In addition, the mature application of laser processing in other high-hardness alloys [3,5,6,7,8,9,10,11,12,20,21,22,23,24,25,26,27,28,29,30] and metal matrix composites [26,31,32,33,34,35,36,37,38] provides a theoretical and technical basis for the fabrication of Fe-Cr-B alloys. (Indirect inference).

As-cast Fe-Cr-B alloys contain more internal cracks and pores than Fe-Si and Fe-Mn cast alloys, which seriously deteriorate wear and corrosion resistance. (Casting). By comparison, laser-clad Fe-B coatings possess fewer defects and uniformly distributed refined borides, showing prominent technical advantages. (Laser cladding). Nevertheless, existing research is relatively scattered: most studies focus on casting [6,24,39] and laser cladding, while systematic investigations on LPBF/DED bulk Fe-Cr-B alloys are insufficient [22]. (Indirect inference). These studies mainly investigate the synergistic effects and wear interaction mechanisms between boride phases (primarily M_2_B) and the metallic matrix. These interactions are affected by solid solution strengthening and crystallographic differences between the two phases, and the boride phases can mitigate harmful interfacial reactions through their physical barrier effect. (LPBF/DED). Chromium (Cr) can improve the fracture toughness of boride phases (e.g., M_2_B) and enhance the wear resistance of the alloys, yet coarse boride phases are prone to induce stress concentration effects. In addition, despite the excellent application potential of Fe-Cr-B alloys and the existence of numerous independent studies on either Fe-Cr-B alloy metallurgy or LAM fabrication process principles, current research is characterized by a scattered distribution, lacking a systematic discussion on the in-depth integration of Fe-Cr-B alloy metallurgy, LAM fabrication process physics, and the resulting performance correlations. There is a lack of systematic connection among Fe-Cr-B alloy metallurgy, laser processing mechanism and service performance correlation. (Indirect inference). This review strictly distinguishes research data from casting, laser cladding and bulk LAM, labels each conclusion with corresponding evidence sources, and establishes a unified theoretical framework of metallurgy–process–microstructure–service performance.

Fe-Cr-B alloys have great potential to break the performance limit of traditional materials by virtue of their high-hardness boride reinforced phases and corrosion-resistant matrix. Laser processing realizes active regulation of boride size, distribution and matrix microstructure through unique non-equilibrium solidification. The cross-integration of alloy design and laser processing promotes the transformation from macroscopic geometric manufacturing to precise microstructure manufacturing, and drives the development of high-performance components toward customization, lightweight and functional integration. Laser-processed Fe-Cr-B alloys can be applied to automotive brake parts, bearings, hot-dip galvanizing sink roll sleeves, hot-rolled guide rolls and other difficult-to-cast and wear–corrosion prone components. (Indirect inference).

Accordingly, this review focuses on the processing–microstructure–performance relationship and systematically summarizes research progress of Fe-Cr-B alloys fabricated by casting, laser cladding, laser remelting, and laser additive manufacturing. (LPBF/DED). All conclusions are strictly categorized by evidence type: LPBF, DED, laser remelting, laser deposition, laser cladding, casting, and indirect inference. Conclusions from laser cladding coatings are not extrapolated to bulk LAM components. Quantitative comparisons of grain size, boride morphology, phase composition, density, hardness, toughness, wear rate, and corrosion current density are conducted. Non-equilibrium solidification mechanisms, including cooling rate differences, solute trapping, undercooling, G/R-dependent CET, and phase precipitation thermodynamics, are deeply discussed. Key bottlenecks including incomplete performance databases, insufficient post-processing optimization, and missing non-equilibrium phase evolution data are clarified. Targeted research routes and standardization strategies are proposed. This review aims to provide a unified theoretical and data reference for controllable preparation and performance-oriented design of laser-processed Fe-Cr-B alloys.

The literature search and inclusion/exclusion criteria are as follows. This review retrieves literature from the Web of Science, Elsevier ScienceDirect, Springer, Scopus, Wiley, Engineering Village, CNKI databases. Core search keywords include the following: Fe-Cr-B alloy, Fe-Cr-B powder, laser additive manufacturing (LAM), 3D printed, laser powder bed fusion (LPBF), directed energy deposition (DED), laser cladding, casting, wear resistance, corrosion resistance, microstructure control, and boride phase. The time span covers 2016–2026. A total of 469 papers were initially retrieved; 212 were screened by title/abstract/key word; 128 were excluded due to irrelevance, lack of experimental data, or non-peer-reviewed sources; and finally, 84 papers were included in this review. Inclusion criteria: peer-reviewed experimental and theoretical studies focused on Fe-Cr-B alloy microstructure, phase evolution and service performance via LPBF, DED, laser cladding and conventional casting. Exclusion criteria: conference abstracts, patents, and pure simulation work without experimental verification.

## 2. Metallurgical Basis and Performance-Determining Factors of Fe-Cr-B Alloy

### 2.1. Phase Diagram and Key Phases

#### 2.1.1. Phase Diagram Calculation and Solidification Process Simulation of Fe-Cr-B Alloys

**Figure 1:**
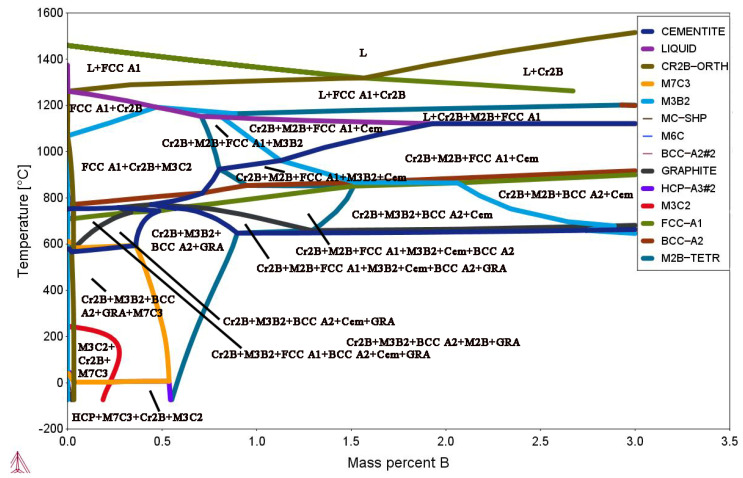
The pseudo-binary vertical section phase diagram of the Fe-Cr-B alloy system calculated by Thermo-Calc [3].

**Figure 2:**
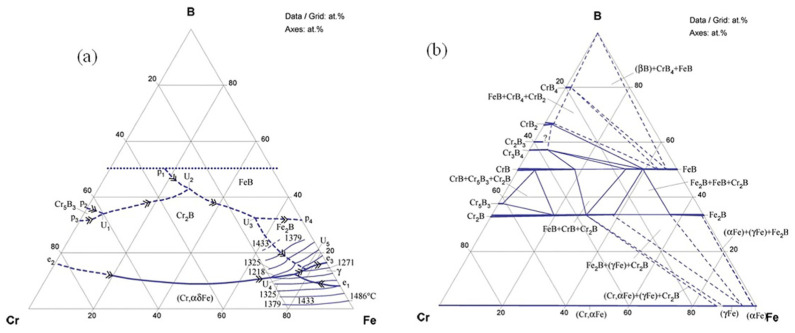
(**a**) Partial liquidus surface projection and (**b**) representative ternary Fe-Cr-B isothermal phase diagram at 900 °C [14].

Taking a Fe-Cr-B alloy with 0.45 wt.% C, 2.0 wt.% Si, 2.2 wt.% Mn, 2.0 wt.% Cr, 0.30 wt.% Mo, 0–3.0 wt.% B, and a small amount of Nb as an example, the pseudo-binary vertical section phase diagram of the Fe-Cr-B alloy system was calculated using Thermo-Calc to reveal the phase precipitation behavior over different B contents, as shown in Figure 1. Different from most previous studies that only adopt pseudo-binary vertical sections, this paper supplements and analyzes the Fe-Cr-B ternary phase diagram (Figure 2), which can comprehensively reveal phase precipitation rules across the full compositional range and provide more accurate thermodynamic basis. During the solidification process, Fe-Cr-B alloy first precipitates Cr_2_B, followed by Fe_2_B, M_2_B, M_3_B_2_, M_7_C_3_, and M_3_C_2_. Finally, the precipitated α-Fe and untransformed RA (γ-Fe) form the metal matrix of the alloy. Among them, M_2_B and M_7_C_3_ are the main hard phases of Fe-Cr-B alloy, which contribute greatly to the improvement of the alloy’s wear resistance. The ordered broken network of small M_2_B boride phases is the key to the improvement of the alloy’s wear resistance [3], while the continuous network boride phases at grain boundaries will degrade the alloy’s wear resistance [14]. (Casting).

#### 2.1.2. Selection of Boride Types

According to the phase diagram characteristics and engineering application demands of Fe-Cr-B alloys, reasonable selection of boride types is essential for composition optimization, aiming to achieve superior wear and corrosion resistance. Numerous studies have confirmed that boron content dominates the type of precipitated borides [6,40,41,42,43]. Fe_3_(B, C) (complex orthogonal structure), Fe_23_(C, B)_6_ (complex face centered cubic structure), M_7_C_3_ (rhombohedral system, hardness up to 1800 HV), Fe_3_C (rhombohedral system, hardness up to 842 HV), Fe_2_B (body centered tetragonal structure, hardness up to 2929 HV), M_2_B (tetragonal system, hardness up to 2653 HV) and TiB_2_ (hexagonal system, hardness up to 3469 HV) are the main hard phases in Fe-Cr-B alloys. All these phases possess excellent high-temperature structural stability up to 1400 °C [3,11,13,16]. The type and morphology of boride phases and the mutual transformation between the boride phases have a great effect on properties of Fe-Cr-B alloys, especially on the fracture toughness and brittleness. For instance, Fe_2_B can form multilayer interfacial structures to pin grain boundaries and restrain grain growth. Owing to its ultra-high hardness, Fe_2_B can significantly strengthen the alloy and improve its comprehensive wear and corrosion resistance. When Fe_2_B maintains a parallel orientation with the α-Fe matrix, the alloy exhibits optimal tribological and anticorrosion performance. Therefore, maximizing the proportion of refined Fe_2_B grains is an effective design strategy for alloy performance optimization. (Casting).

TiB_2_ acts as a critical secondary reinforcing phase in Fe-Cr-B alloys. It can serve as heterogeneous nucleation sites to refine M_2_B grains, form coherent interfacial bonding with the matrix, and synergistically enhance the hardness and wear resistance together with M_2_B relying on its ultra-high hardness (3469 HV). Compared with the Ti-free counterpart, the alloy with TiB_2_ addition shows a 13% increase in hardness and a 58% improvement in impact toughness [11]. (Casting).

### 2.2. Microstructure-Property Relationships of Cast Fe-Cr-B Alloys

This section establishes a generalized microstructure-property correlation for Fe-Cr-B alloys covering diverse compositional systems, rather than being limited to a single specific alloy component. It systematically discusses the effects of boride morphology, matrix constitution (martensite, austenite, bainite) and interfacial characteristics on the hardness, toughness, wear and corrosion resistance of cast Fe-Cr-B alloys. The microstructural morphology, mechanical properties and service performance can be comprehensively characterized via metallographic microscopy, scanning electron microscopy (SEM), transmission electron microscopy (TEM), X-ray diffraction (XRD), Rockwell hardness testing, impact testing and tribological testing. Quantitative and qualitative correlation models are further established to clarify the relationship between microstructural features (boride size, distribution and morphology; matrix type; interfacial structure) and macroscopic mechanical and service properties.

**Figure 3:**
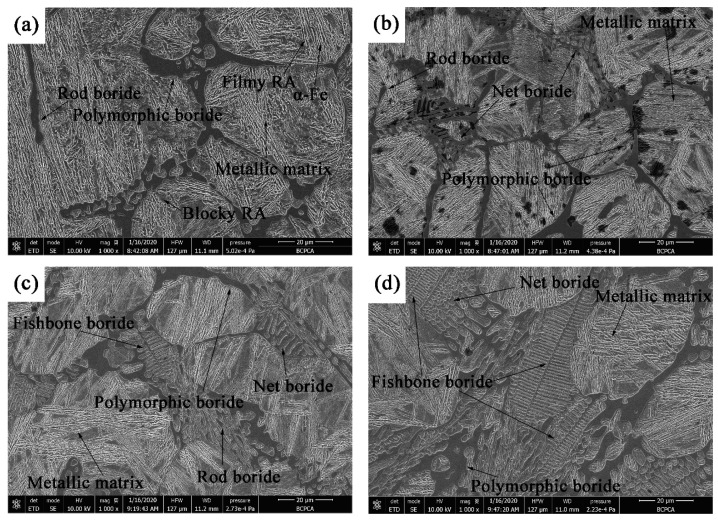
The SEM of as-cast Fe-Si-Mn-Cr-B alloys with different B contents: (**a**) 0.5%; (**b**) 1.0%; (**c**) 1.5%; (**d**) 2.0% [3].

**Figure 4:**
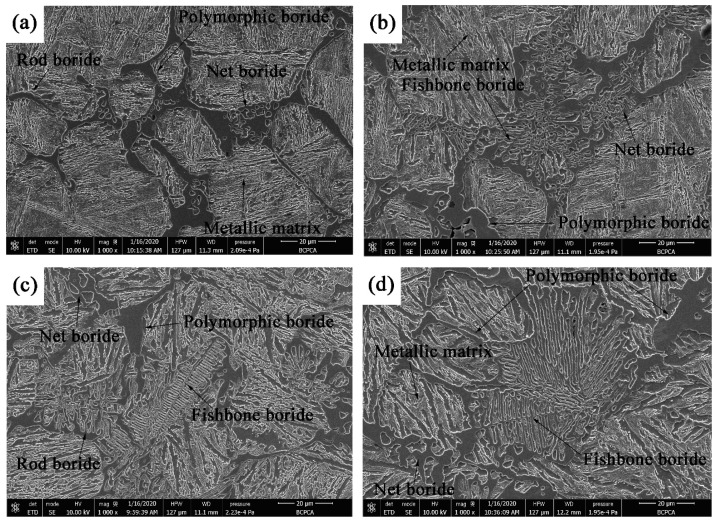
The SEM of normalized Fe-Si-Mn-Cr-B alloys with different B contents: (**a**) 0.5%; (**b**) 1.0%; (**c**) 1.5%; (**d**) 2.0% [3].

A typical alloy with 0.45 wt.% C, 2.0 wt.% Si, 2.2 wt.% Mn, 2.0 wt.% Cr, 0.30 wt.% Mo and a small amount of Nb was selected as the research prototype. Microstructural evolution can be regulated by adjusting boron addition content. The microstructural morphologies of as-cast and normalized Fe-Cr-B alloys with varying boron contents are presented in Figure 3 and Figure 4, respectively. Table 1 is supplemented to quantitatively compare grain size, boride fraction, hardness and toughness of as-cast alloys with different boron contents, replacing previous vague qualitative descriptions. It is observed that when the B content exceeds 1.0 wt.%, a large number of small and uniformly distributed fishbone like boride phases appear in the microstructure of the alloy, and the number of boride phases significantly increases. The metal matrix structure and boride phase gradually refine. After heat treatment, the structure is slightly refined, and the distribution of boride phases is more uniform with a slight increase in quantity. The number of broken mesh and fishbone boride phases significantly increases, reducing sources of stress concentration. Boride phases are mainly embedded in the metal matrix in the form of a fishbone, mesh, rod or irregular polygon, which can isolate and protect metallic grains and form a rigid skeleton during the wear process [14,16]. Such structural features fully combine the high hardness of borides and the good toughness of the matrix, endowing the alloy with excellent wear resistance. Nevertheless, multiphase stacking in the alloy tends to induce crack deflection behavior.

**Figure 5:**
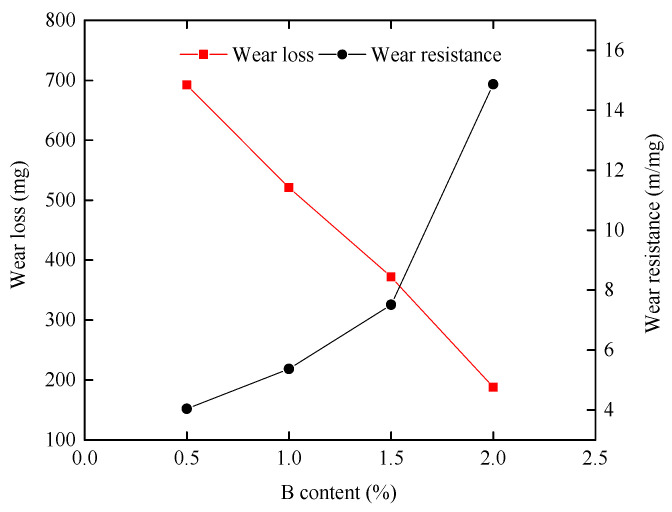
Effect of B content on the wear loss and wear resistance of Fe-Cr-B alloys [3].

**Figure 6:**
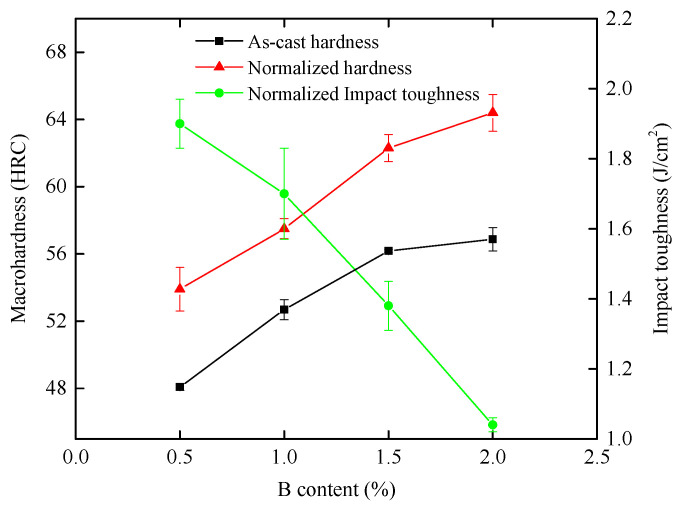
Effect of B content on the macrohardness and impact toughness of Fe-Cr-B alloys [3].

The fracture surface is dominated by large cleavage planes with a small number of ductile dimples and microcracks, indicating a typical brittle fracture mode. Alloys with higher boron content exhibit larger cleavage planes, fewer ductile dimples and more interfacial microcracks, which originates from the high-volume fraction of brittle boride phases. Comparative experiments show that when the B content increases from 0.5 wt.% to 2.0 wt.%, the boride fraction rises by 13%, while the impact toughness declines by 45% (Figure 5 and Figure 6). This phenomenon is attributed to the poor capacity of high-B alloys to absorb impact energy, passivate crack tips, and hinder crack initiation and propagation [3]. By contrast, the alloy with 0.5 wt.% B possesses numerous fine dimples on the fracture surface due to the high fraction of α-Fe and retained austenite (RA), thereby maintaining superior toughness. Further analysis is conducted to clarify the inherent correlation between microstructure and macroscopic properties including hardness, toughness, wear and corrosion resistance.

**Figure 7:**
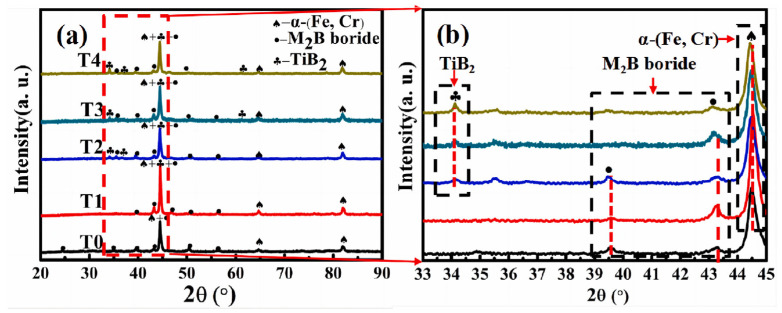
TiB_2_ diffraction peak enhancement: (**a**) original image; (**b**) Partial enlarged view [11].

The overall hardness of Fe-Cr-B alloys is predominantly determined by the type, morphology, volume fraction, size and distribution uniformity of hard boride phases such as M_2_B and TiB_2_. These hard phases enhance their resistance to plastic deformation by hindering dislocation motion. The introduction of TiB_2_ can refine Cr-rich M_2_B grains and transform coarse networked M_2_B into uniformly dispersed discrete particles. Meanwhile, TiB_2_ and M_2_B jointly form a diffusion barrier to suppress excessive precipitation and aggregation of borides, achieving an optimal balance between boride hardness and matrix toughness. This strengthening effect is mainly derived from the dispersion strengthening of TiB_2_ and grain refinement of M_2_B. As illustrated in Figure 7, compared with the Ti-free T0 alloy, the T3 alloy with TiB_2_ addition exhibits increased boride content and achieves a 13% hardness enhancement [11].

**Figure 8:**
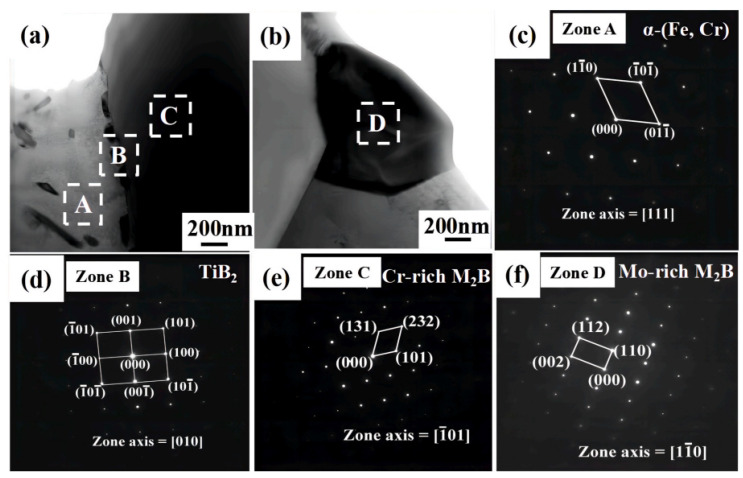
The interface between TiB_2_ and the matrix is coherent, resulting in no microcracks at the interface: (**a**,**b**) Bright-field micrographs and (**c**–**f**) corresponding SADPs of the matrix and borides; (**c**) α-(Fe, Cr); (**d**) TiB_2_; (**e**) Cr-rich M_2_B boride and (**f**) Mo-rich M_2_B boride [11].

**Figure 9:**
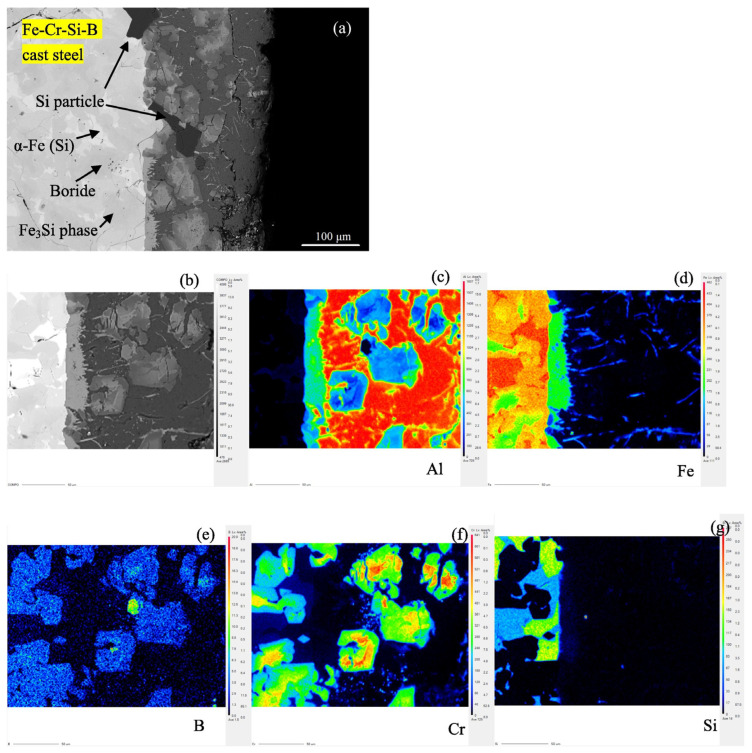
Crack deflection induced by the interface: (**a**) cross-sectional morphologies after HDA, (**b**) elemental map analysis of cross-sectional morphologies after HDA. Coincidentally, the primary Si phase did not appear in (**b**), so (**a**) was used to comprehensively illustrate the interfacial microstructure of the Fe–Cr–B–Si cast steel after HDA, (**c**–**g**) are the elemental maps for Al, Fe, B, Cr, and Si, respectively [16].

Toughness reflects the ability of a material to absorb energy and resist crack propagation, which is determined by the size and distribution of hard phases, the interfacial bonding strength between hard phases and the matrix, process-induced porosity, and crack deflection mechanisms. As shown in Figure 8 and Figure 9, the TiB_2_ phase refines M_2_B and enhances interfacial bonding, and the corrosion layer thickness is ~20–40 μm. The TiB_2_-matrix interface and the discrete distribution of boride phases reduce stress concentration, and Si particles and the interface with borides induce crack deflection or suppress crack initiation, resulting in an impact toughness of 32.4 kJ/m^2^ in T3 alloy, which is 58% higher than that of T0 alloy [11,17]. Cracks propagate along the boride/matrix interface and deflect.

**Figure 10:**
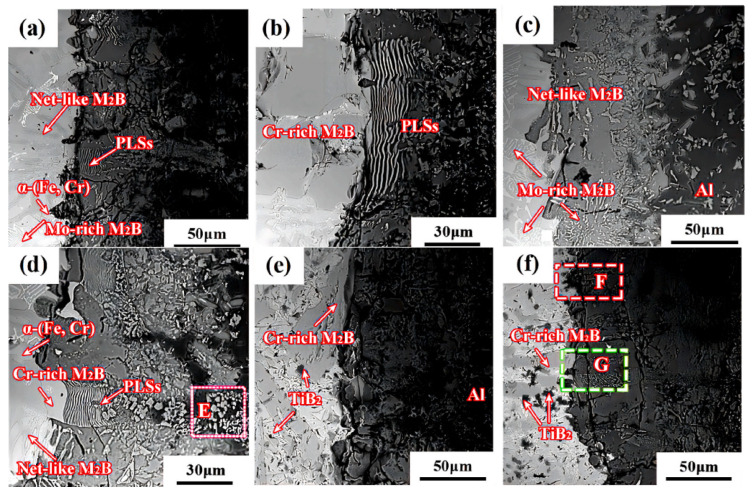
Smooth wear surface formed by the synergy of TiB_2_ and M_2_B: (**a**,**b**) T0 sample, (**c**,**d**) T1 sample, (**e**,**f**) T2 sample [11].

Wear resistance is a synergistic effect of hardness and toughness, governed by the wear resistance of hard phases and the plastic deformation capacity of the matrix. It is also influenced by the environment, and the microstructure of the alloy affects the wear mechanism, such as micro cutting, oxidative wear, and fatigue spalling. Among them, high-hardness M_2_B boride phases directly resist abrasive wear, TiB_2_ reduces adhesive wear, and α-Fe provides plastic buffering to prevent boride detachment. In the dry friction test, the high-hardness alloy exhibited the lowest wear rate (8.16 × 10^−7^ mm^3^/Nm) because boride phases hinder the penetration of abrasive particles. However, in water friction, the medium-hardness alloy performs the best, as the matrix effectively supports the boride framework [17]. As shown in Figure 10, wear layer thickness is 5–15 μm. TiB_2_ and M_2_B synergistically form a diffusion barrier, mitigating abrasive cutting, but pores and oxides act as stress concentration points, serving as crack initiation sources and accelerating wear [11]. Therefore, to achieve ideal wear and corrosion resistance, small, isolated boride particles should be uniformly embedded in a martensite/austenite matrix, and the martensite/austenite ratio should be adjusted to an appropriate level (toughening matrix) to simultaneously provide good toughness and high wear resistance.

**Figure 11:**
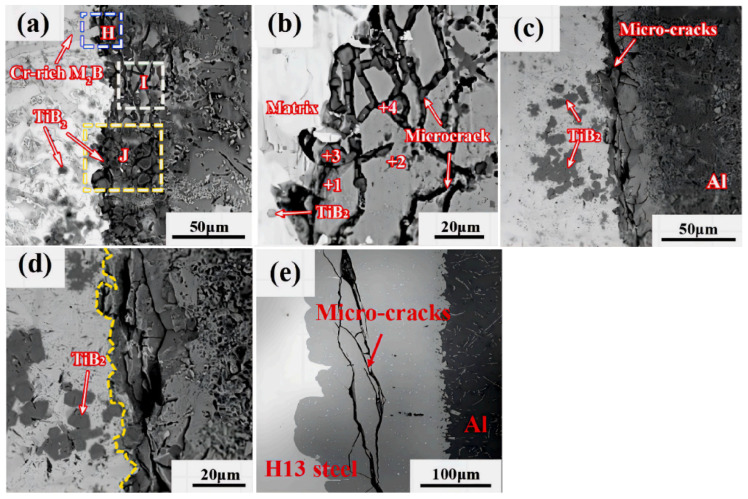
Periodic layered structure formed by TiB_2_ and M_2_B corrosion products: (**a**,**b**) T3 sample, (**c**,**d**) T4 sample, (**e**) H13 steel [11].

Corrosion resistance depends on phase interface stability, elemental distribution, passivation film formation ability, interphase galvanic corrosion tendency and elemental diffusion barrier. The corrosion mechanism of Fe-Cr-B alloys is mainly divided into selective corrosion (cast alloys) and pitting corrosion (LAM/laser cladding alloys). Chromium (Cr) is the core element for corrosion resistance: it forms a dense Cr_2_O_3_ passive film on the alloy surface, blocking the penetration of corrosive media; the boride/matrix interface is the key area for corrosion initiation. Continuous networked borides form galvanic cells with the matrix, accelerating corrosion, while refined, dispersed borides reduce the driving force for galvanic corrosion [12,16,17]. The corrosion products of TiB_2_ and M_2_B form a periodic layered structure, which further enhances the stability of the passive film (Figure 11) [16]. TiB_2_ significantly shortens the crack propagation length and hinders the crack from propagating along the network boride, and the crack is nailed and terminated by TiB_2_ particles. The crack length is 5–15 μm, and the corrosion layer thickness is ~10–25 μm.

### 2.3. Corrosion Process, Corrosion Mechanism, and Action Mechanism of Phase Potential Difference and Precipitates in Fe-Cr-B Alloys

In addition to wear failure, corrosion and tribo-corrosion coupled failure are dominant service failure forms of Fe-Cr-B alloys. The corrosion behavior is collectively governed by the multiphase heterogeneous microstructure, boride precipitation characteristics, and potential difference between various phases [6,29].

#### 2.3.1. Evolution Process of Corrosion (Casting)

**Figure 12:**
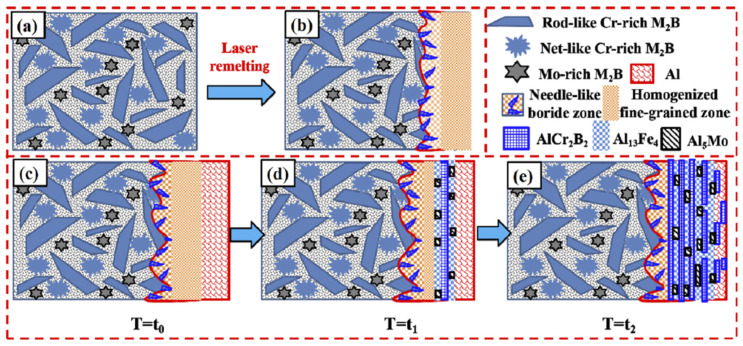
Corrosion mechanism of the laser-remelted Fe-Cr-B-Mo alloy layer: (**a**) as-cast Fe-Cr-B-Mo alloy; (**b**) Laser-remelted Fe-Cr-B-Mo alloy; (**c**–**e**) visual schematic diagram of the corrosion process: (**c**) Initial state (T = t_0_), (**d**) Intermediate states (T = t_1_), and (**e**) Final state (T = t_2_) [10].

Under service conditions such as salt solution, acid–alkali medium, and high-temperature molten aluminum, Figure 12 intuitively presents the corrosion evolution process of Fe-Cr-B alloys of the remelted layer. It visually compares the corrosion path of continuous network borides and refined discrete borides, providing clear graphical support for corrosion mechanism interpretation.

(1) Oxygen-absorption corrosion activation stage: After the corrosive medium contacts the alloy surface, the α-(Fe,Cr) matrix with lower corrosion potential preferentially undergoes anodic dissolution, and a thin, unstable initial oxide film composed of FeO, Fe_2_O_3_ and Cr_2_O_3_ rapidly forms on the surface [10,22].
(2) Initiation and propagation of micro-galvanic corrosion: High-potential and homogeneous fine-grained M2B-type borides form a large number of micro-galvanic couples (solid–liquid interface diffusion couple) with the matrix, as shown in Figure 12c. The matrix acts as the anode for continuous dissolution, and corrosion preferentially propagates along phase interfaces and grain boundaries [10,22].
(3) Passive film rupture and localized corrosion development: After a period of corrosion, Cr elements enrich on the surface to form a Cr_2_O_3_-rich passive protective film, or between Cr-rich M2B borides and Al, or between Mo-rich M2B borides and Al, as shown in Figure 12d. However, network-shaped borides destroy the integrity of the passive film; aggressive ions such as Cl^−^ are easily adsorbed at phase interfaces and induce passive film breakdown, further leading to pitting corrosion, intergranular corrosion and groove corrosion [6,10].
(4) Accumulation of corrosion products and steady-state corrosion: A double-layer structure forms on the alloy surface, consisting of a dense Cr-rich inner oxide layer and a loose outer composite layer of iron oxyhydroxides and borates. The loose products readily dissolve and peel off, allowing continuous penetration of corrosive media and driving corrosion into a steady-state propagation stage, as shown in Figure 12e [6,10].

Under the coupled conditions of high-temperature molten aluminum and friction, corrosion is accompanied by elemental interdiffusion, intermetallic compound formation and mechanical spalling acceleration, resulting in a more complex failure mechanism [10,17,22]. (Indirect inference).

#### 2.3.2. Main Corrosion Types and Coupled Corrosion Mechanisms

The main corrosion types of Fe-Cr-B alloys include micro-galvanic corrosion, intergranular corrosion, pitting corrosion, groove corrosion, molten aluminum interfacial corrosion, and tribo-corrosion synergistic corrosion. The corrosion behavior is controlled by the coupling of multiple mechanisms rather than a single one [6,17].

(1) Dominant micro-galvanic corrosion mechanism

A significant potential gradient exists among the multiphase microstructure, with the potential sequence: Mo-rich M_2_B > (Fe,Cr)_2_B > Fe_2_B > α-Fe matrix. The potential difference between phases can reach hundreds of millivolts. A larger cathode area fraction of borides leads to a stronger driving force of micro-galvanic corrosion. Continuous network borides in as-cast alloys easily form large-scale cathode regions and significantly accelerate the anodic dissolution of the matrix [29].

(2) Passivation–depassivation competitive mechanism

Cr is the core passivation element of Fe-Cr-B alloys. Solid-solution Cr can form a dense and stable Cr_2_O_3_ passive film. Nevertheless, the precipitation of B consumes the effective Cr content in the matrix; excessive Cr tends to induce nanoscale elemental segregation and form alternating Cr-rich and Cr-poor zones, destroying the uniformity of the passive film. Molybdenum participates in the reconstruction of the passive film by generating MoO_4_^2−^, inhibiting the dissolution of Cr ions and further improving film stability [29].

(3) Grain boundary segregation corrosion mechanism

During solidification, B, Cr and Mo tend to undergo non-equilibrium segregation at grain boundaries and form network grain-boundary borides, resulting in inhomogeneous composition and potential distribution at the grain boundaries. This promotes rapid inward corrosion propagation along grain boundaries and induces intergranular cracking and layered spalling [6] (casting).

(4) Interfacial reaction mechanism in molten aluminum

M_2_B borides can undergo metallurgical reactions with molten aluminum to form intermetallic compounds such as AlCr_2_B_2_ and Al_5_Mo, and further develop periodic layered structures (PLSs), which effectively hinder the interdiffusion of Al atoms. Nevertheless, mechanical damage induced by high rotating speed and high load can destroy the layered structure, induce microcracks and accelerate the spalling of corrosion products [10] (Laser remelting).

(5) Tribo-corrosion coupling mechanism

Mechanical loading continuously scrapes off the passive film and corrosion products, exposing fresh matrix and accelerating electrochemical dissolution. Meanwhile, corrosion weakens the interfacial bonding strength between the matrix and borides, aggravates hard phase fracture and abrasive wear, and forms a synergistic failure cycle in which corrosion accelerates wear and wear promotes corrosion [17] (Indirect inference).

#### 2.3.3. Regulation Law of Phase Potential Difference and Precipitates on Corrosion Resistance (Casting)

The magnitude of phase potential difference directly determines the intensity of micro-galvanic corrosion. A larger potential difference between the matrix and Fe_2_B/(Fe,Cr)_2_B corresponds to a higher kinetic rate of anodic matrix dissolution. Modifying borides via Cr and Mo solid solution substitution can effectively reduce the phase potential difference and weaken the micro-galvanic effect [29].

The type, morphology, distribution and volume fraction of precipitates exert decisive effects on corrosion behavior:

(1) Precipitate type: Fe_2_B possesses high brittleness and large phase potential difference, leading to the strongest micro-galvanic corrosion effect. (Fe,Cr)_2_B and Cr_2_B exhibit well-matched potential with excellent passivation ability. Mo-rich M_2_B shows improved high-temperature stability and superior molten aluminum corrosion resistance. M_7_B_3_ composite borides have good interfacial compatibility and are less prone to induce groove corrosion [6,10].
(2) Morphology and distribution: Network continuous borides destroy the integrity of the matrix and form penetrating corrosion channels, making it difficult to form a continuous passive film and thus deteriorating corrosion resistance significantly. Dispersed granular borides have no continuous corrosion pathways with limited galvanic effect, facilitating the formation of intact passive films and achieving optimal corrosion resistance. Coarse dendritic borides at grain boundaries aggravate elemental segregation and preferentially induce intergranular corrosion and pitting corrosion [6].
(3) Volume fraction: Excessively high-volume fraction of borides sharply increases the cathode area ratio and raises the micro-galvanic current density greatly. Laser non-equilibrium solidification can realize refined and dispersed precipitation of borides, which not only weakens the galvanic effect of individual phases but also refines grains and enhances the overall passivation ability [33].

In summary, the key to achieving ideal comprehensive performance of Fe-Cr-B alloys is to realize a “fine, isolated hard-phase distribution + strong, tough matrix” structure.

## 3. Casting of Fe-Cr-B Alloys: Challenges and Optimization Strategies

The excellent wear resistance of Fe-Cr-B alloy mainly originates from the high-hardness boride hard phases distributed within the matrix. However, during the casting solidification process, if the composition or process control is improper, defects such as cracks, pores, and coarse boride phases are easily generated, which severely degrade the alloy’s toughness and reliability. The reason is that there are many alloying elements in the organization, and element segregation occurs under slow cooling conditions during casting. The severe segregation of boron, the coarsening and growth of boride phases, and the accumulation of thermal stress are the root causes of poor toughness and high crack susceptibility. In addition, the variety and high-volume fraction of borides lead to microstructural inhomogeneity. Therefore, modifiers are usually added during casting to improve the alloy microstructure, but significant defects still persist.

### 3.1. Main Internal Casting Defects and Their Formation Mechanisms of Fe-Cr-B Alloys

Coarse boride phases with a continuous network distribution are the most critical internal casting defects in Fe-Cr-B alloys. When the B content exceeds its solubility limit in the iron matrix, boride phases will be formed preferentially, such as (Cr,Fe)_2_B. Under the slow cooling rate of conventional casting, boride phases have sufficient time to grow up and tend to precipitate along the grain boundary, forming thick lath-like, fishbone-like, or continuous network structures, as shown in Figure 2 [3]. These coarse, brittle borides act like “hard inclusions” inside the alloy; they have sharp morphologies and weak bonding with the ductile matrix. Under external or thermal stress, they become stress concentration points, and cracks are easily initiated at this interface and propagate along the boride network. This significantly increases alloy brittleness and reduces impact toughness. In addition, during the wear process, continuous network boride phases are prone to overall fracture and peeling due to their brittleness, rather than gradual wear, resulting in reduced wear resistance.

Cracks are usually not independent defects, but rather consequences caused by other defects, particularly coarse borides. During the slow cooling process of casting, significant internal stresses are generated due to the difference in thermal expansion coefficients between boride phases and the matrix. The stress concentration effect caused by coarse boride phases will exacerbate this internal stress; when the stress exceeds the material’s strength limit, hot cracks form. In addition, as described in references, volume changes caused by subsequent heat treatment processes such as thermal diffusion treatment, phase transformation, such as the formation of FeAl_2_O_3_, and element interdiffusion, such as the dissolution of Fe atoms from (Cr,Fe)_2_B, can also induce microcracks around the original coarse borides [27,39].

Pores mainly originate from gases entrapped during melting and pouring, or gases generated by reactions between mold moisture and molten metal. The presence of pores disrupts matrix continuity, serves as crack initiation sources, and reduces the alloy’s density and effective load-bearing area.

### 3.2. Improvement Methods and Measures

To mitigate the aforementioned casting defects and performance limitations, modifier treatment and post-heat treatment have been widely investigated.

#### 3.2.1. Modification Processing

The core purpose of modifier microalloying is to tailor the morphology, size and distribution of borides, transforming continuous brittle networks into isolated fine particles or short rod-like precipitates. For wear-resistant cast alloys, the total addition of alloying elements including Silicon (Si), Manganese (Mn), Cr, Molybdenum (Mo), Nickel (Ni) and Niobium (Nb) is generally controlled below 5 wt.% to minimize casting defects and improve microstructural homogeneity [40,41]. Combined with previous experimental results and Thermo-Calc thermodynamic calculations [3,13,42,43], a basic alloy composition containing 0.45 wt.% Carbon (C), 2.0 wt.% Si, 2.2 wt.% Mn, 2.0 wt.% Cr, 0.30 wt.% Mo and a small amount of Nb is adopted for microalloying refinement. Microstructural and mechanical properties can be further optimized by adjusting B content. The functions of major alloying elements are summarized as follows:

(1) Carbon improves alloy hardenability and provides solid solution strengthening, acting as a decisive element for balancing hardness and toughness. Excess carbon leads to loose microstructures and degraded toughness, while carbon content exhibits limited influence on boron carbide fraction. Specifically, every 0.1 wt.% increase in carbon content raises boron carbide volume fraction by approximately 1%. Low carbon content enhances toughness at the expense of hardness and wear resistance.
(2) Niobium is a strong carbonitride-forming element. It pins austenite grain boundaries to suppress grain growth and provides favorable conditions for ferrite refinement. Strain-induced carbonitride precipitation inhibits the growth and recrystallization of deformed grains, achieving simultaneous grain refinement and precipitation strengthening.
(3) Boron exhibits extremely low solid solubility in iron matrices, forming substitutional solid solutions in α-Fe and interstitial/substitutional solid solutions in γ-Fe. As the dominant alloying element, boron governs the formation of hard boride and boron carbide phases, and its influence on alloy properties is 1.7 times that of carbon. Insufficient boron fails to form adequate wear-resistant hard phases; excessive boron promotes the precipitation of coarse brittle borides and deteriorates impact toughness.
(4) Chromium is a typical ferrite-forming and weak carbide-forming element. Cr addition does not change boride types but modifies their morphology and distribution, breaks continuous boride networks, and enhances the hardness and fracture toughness of M_2_B phases. Low Cr content favors Fe-rich tetragonal M_2_B, while Cr content above 15 wt.% promotes the transformation to high-toughness orthogonal (Cr,Fe)_2_B. Meanwhile, Cr forms a dense Cr_2_O_3_ oxide film at high temperature (the melting point of Cr is 1903 °C), improving oxidation resistance and fretting wear performance [14].
(5) Silicon is a non-carbide and ferrite-forming element with strong oxygen affinity. It provides solid solution strengthening and deoxidation effect, reduces the austenite phase region, and suppresses cementite coarsening. Appropriate Si addition promotes the formation of dense Cr_2_O_3_/SiO_2_ composite passive films; excessive Si addition induces matrix embrittlement.
(6) Manganese is an austenite-stabilizing element that expands the austenite phase region and increases the solubility of Cr and C. Moderate Mn addition refines pearlite microstructures; excessive Mn increases retained austenite content and reduces wear resistance. Moreover, Si and Mn can dissolve into the matrix to strengthen the substrate and inhibit excessive boride growth, facilitating the formation of fragmented fishbone-like boride structures [22].
(7) Molybdenum acts as an effective solid-solution strengthening element [44]. It increases recrystallization temperature, refines grain size, and stabilizes austenite during cooling. Mo addition modifies boride dendritic morphology and increases the volume fraction of carboboride phases, thereby enhancing alloy hardness and wear resistance [14,22]. Research [14] further states that an increase in Mo content will increase the volume fraction of carboboride phases, thereby improving hardness. When the Mo content is 25% and 30%, the wear resistance is better [45]. Nevertheless, Mo is a high-cost precious alloying element [46].

Microalloying with the above elements can effectively tailor the morphology, size and distribution of precipitates, optimizing the microstructure and comprehensive performance of Fe-Cr-B alloys [16,47,48].

#### 3.2.2. Heat Treatment

Heat treatment is a key post-processing step to improve casting structure, eliminate internal stress, and enhance overall performance.

Normalization and homogenization annealing: A study [22] has shown that after 900 °C air-cooled normalizing treatment, the distribution of boride phases becomes more uniform, and the broken network structure is more obvious, as shown in Figure 4 and Figure 5. The macroscopic hardness of the alloy is significantly improved, as shown in Figure 8, and the wear resistance is significantly enhanced. The mechanism is to redistribute the elements through heating and insulation and use the relatively fast cooling rate of air cooling to suppress the re-coarsening of boride phases, making boride phases become small broken mesh or granular, reducing stress concentration, increasing crack propagation resistance, and thus improving the impact toughness of the alloy. At the same time, high-hardness bainite and other matrix structures are obtained, and the synergistic effect of high-hardness boride phases and strong matrix is enhanced.

Thermal diffusion treatment (TDT): Given that the intense interdiffusion of elements during the TDT process leads to significant changes in the microstructure [27,39,49], appropriate high-temperature diffusion annealing alleviates elemental segregation, passivates sharp boride edges, and promotes boride spheroidization [14].

Overall, the defect control of as-cast Fe–Cr–alloys is a systematic engineering problem. The core bottleneck originates from coarse continuous network borides, which raise crack sensitivity and degrade toughness. Effective solutions include precise regulation of B and alloying element contents, optimization of boride morphology via Cr/Si/Mo microalloying, and adoption of normalization annealing to relieve residual stress and promote boride spheroidization. Extreme non-equilibrium solidification and powder metallurgy can fundamentally eliminate inherent casting defects and achieve ultrafine ideal microstructures. Comprehensive adoption of the above strategies enables Fe-Cr-B alloys to maintain excellent wear resistance while possessing sufficient toughness for harsh industrial service conditions. (Indirect inference).

## 4. Laser Additive Manufacturing of Fe-Cr-B Alloys: A Paradigm Shift

Based on the above discussion, breaking the limitation of traditional equilibrium solidification kinetics is the key to fully releasing the performance potential of Fe-Cr-B alloys. Laser additive manufacturing (LAM) with inherent extreme non-equilibrium rapid solidification characteristics provides a practical approach. (Indirect inference). It should be emphasized that microstructure and performance conclusions obtained from laser cladding cannot be directly applied to LPBF/DED bulk alloys. Meanwhile, the solidification path and defect mechanism of laser cladding are not equal to bulk LAM. Under optimized processing parameters, the relative density of LAM-fabricated bulk Fe-Cr-B alloys can exceed 98%, much higher than that of conventional cast counterparts [22]. (DED). LAM produces fine equiaxed grains [48], and residual void defects are closely related to process parameters and can be minimized via parameter optimization. Microstructural refinement is the dominant approach to enhance mechanical performance. In addition, subsequent heat treatment can further tailor the morphology, size and spatial distribution of constituent phases and borides, achieving further microstructure and performance improvement. Surface roughness can also be optimized by adjusting LAM parameters or implementing post-physical and chemical treatment [2,50]. Nevertheless, process-induced porosity, particle stacking and printing anisotropy still affect the performance consistency of LAM metal matrix composites [51]. Layer-by-layer deposition leads to obvious mechanical anisotropy and poor interlayer bonding, which are closely associated with the inherent thermal cycling characteristics of LAM. (Laser deposition). This section focuses on the metallurgical essence of LAM extreme non-equilibrium rapid solidification, and further discusses its application prospects and existing challenges.

### 4.1. Unique Microstructure Induced by Extreme Non-Equilibrium Rapid Solidification

This article strictly defines three types of processes: traditional casting, laser cladding, and laser additive manufacturing (LAM/LPBF/DED), to avoid conceptual confusion: laser cladding belongs to surface-modified coating technology, and many of the existing microstructure and performance rules are indirect research inferences, which cannot be directly equated with bulk LAM materials; laser additive manufacturing relies on the extreme non-equilibrium layer by layer solidification characteristics of lasers, which can achieve overall grain homogenization of bulk materials, and the related microstructure evolution and performance conclusions have direct experimental support (LPBF/DED); and traditional casting is characterized by near-equilibrium slow solidification, which easily generates coarse and continuous network borides, and has many structural defects. The corrosion and wear mechanisms are fundamentally different from those of laser processed alloys. The following text discusses the microstructure, corrosion and wear behavior, and mechanism separately according to three types of processes, strictly distinguishing between direct experimental conclusions and indirect derivation rules from the literature [10,17,32].

LAM is essentially an extreme non-equilibrium metallurgical process, and its microstructural evolution is dominated by ultra-high temperature gradient (*G*) and solidification rate (*R*) [29,30,52,53]. Previous studies overly generalized cooling rate ranges; this paper subdivides and clarifies specific cooling rates, and analyzes data uncertainty and parameter-induced variability. (Indirect inference). Conventional casting undergoes slow cooling at 10^−1^~10^2^ K/s under near-equilibrium conditions. In contrast, the high-energy laser beam induces instantaneous melting and rapid cooling of the molten pool at the millisecond-to-microsecond scale during LAM, which is equivalent to an ultra-fast quenching process. (Indirect inference). The cooling rate ε, defined as the product of *G* and *R*, reaches 10^3^~10^6^ K/s for laser cladding and 10^6^~10^8^ K/s for LPBF/DED [3,38]. Such ultra-high cooling rates completely alter the thermodynamic and kinetic conditions of alloy solidification and induce distinctive microstructural evolution [54].

In terms of nucleation and grain growth, ultra-high cooling rates produce enormous thermal undercooling (ΔT), constitutional undercooling (ΔT_c_), curvature undercooling (ΔT_R_) and kinetic undercooling (ΔT_K_). The combined undercooling significantly increases nucleation driving force and nucleation density. Meanwhile, the ultra-fast advancing solidification interface severely restricts atomic diffusion and interface relaxation, suppressing subsequent grain growth. Consequently, the coarse columnar/equiaxed grains (hundreds of micrometers to millimeter scale) in cast alloys are transformed into micron/submicron cellular and dendritic grains, or even featureless grains under extremely high solidification rates. At high scanning speeds, high-density dislocations and nano-twins form inside grains, further refining microstructural units [55,56]. (Indirect inference).

According to CET (columnar-to-equiaxed transition) theory and growth restriction factor (GRF) mechanisms, grain morphology during solidification is governed by the G^n^/R **ratio** and solute-driven undercooling [1,56,57,58]. Under the extremely high thermal gradients and rapid solidification rates of LPBF/DED, constitutional undercooling is weakened, while thermal undercooling, curvature undercooling, and kinetic undercooling dominate [1,57].

In terms of solute distribution and segregation, extreme non-equilibrium solidification achieves microstructural homogenization via two dominant mechanisms [57,58], which come from laser cladding (Laser cladding/Indirect inference):

(1) Interface absolute stability theory: When the solidification front velocity exceeds solute diffusion velocity, the solid–liquid interface becomes stable, restraining dendritic growth and forming segregation-free planar or cellular structures.
(2) Solute trapping effect: Under extreme non-equilibrium conditions, solute atoms such as B and Cr are trapped by the rapidly advancing solidification front and frozen in the solid phase without sufficient time for liquid-phase diffusion.

This effect greatly alleviates interdendritic microsegregation and improves compositional homogeneity. Meanwhile, the solid solubility of B and Cr in the α-Fe matrix is remarkably enhanced beyond the prediction of equilibrium phase diagrams, which strengthens the matrix via solid solution strengthening and thermodynamically restrains the precipitation of coarse continuous boride networks, such as (M,Fe)_2_B [57,58]. (Laser cladding/Indirect inference).

In terms of phase transformation pathways, suppressed atomic diffusion and high undercooling enable the alloy to deviate from the equilibrium phase transition sequence. When the alloy composition falls within the glass-forming range and the cooling rate is sufficiently high, long-range atomic diffusion is inhibited, and amorphous structures form directly without crystallization [1,34]. Even for crystalline alloys, the type, morphology and distribution of precipitated phases deviate significantly from equilibrium cast microstructures. (Indirect inference).

The essential advantage of LAM lies in breaking the diffusion-controlled phase transformation rule via ultra-high non-equilibrium solidification kinetics (high G and high R). The most prominent microstructural feature is that the traditional tens-to-hundreds micron continuous network borides are refined by 1–2 orders of magnitude into submicron/nano isolated spherical or short rod-like reinforcing particles dispersed in the supersaturated matrix (indirect inference only, from laser cladding, not applicable to bulk LAM directly). Such a transition from continuous brittle skeleton to dispersed strengthening particles lays the fundamental material basis for LAM to simultaneously optimize the hardness, toughness, wear and corrosion resistance of Fe-Cr-B alloys.

### 4.2. Laser Cladding of Fe-Cr-B Alloy Coatings: Only as Precursor Technology

According to metal solidification theory, grain refinement can be realized by increasing heterogeneous nucleation sites, elevating nucleation rate, restraining nucleus growth, and optimizing front temperature field distribution. Grain refinement can simultaneously enhance strength, hardness, plasticity and toughness, reduce casting shrinkage and second-phase size, and minimize internal defects. It also contributes to the improvement of corrosion resistance, wear resistance and machinability. Therefore, most existing studies adopt grain refinement as the core strategy to optimize the microstructure and comprehensive performance of wear-resistant and high-temperature alloys. (Indirect inference).

As an important branch of LAM technology, laser cladding is a surface modification technology, which deposits dense metallurgical coatings layer by layer via high-energy laser fusion of alloy powder on substrate surfaces [2,25]. In recent years, laser cladding has been widely applied to prepare Fe-Cr-B alloy coatings. It can significantly refine microstructures, enhance coating–substrate metallurgical bonding, and thus improve hardness, wear resistance and corrosion resistance. This section systematically elaborates how laser cladding eliminates the inherent defects of cast Fe-Cr-B alloys through microstructural refinement and interfacial optimization. However, all relevant conclusions belong only to surface coating materials, and cannot be extrapolated to bulk structural parts. Laser remelting further optimizes interfacial structure and passive film stability.

#### 4.2.1. Microstructure Refinement Mechanism

**Figure 13:**
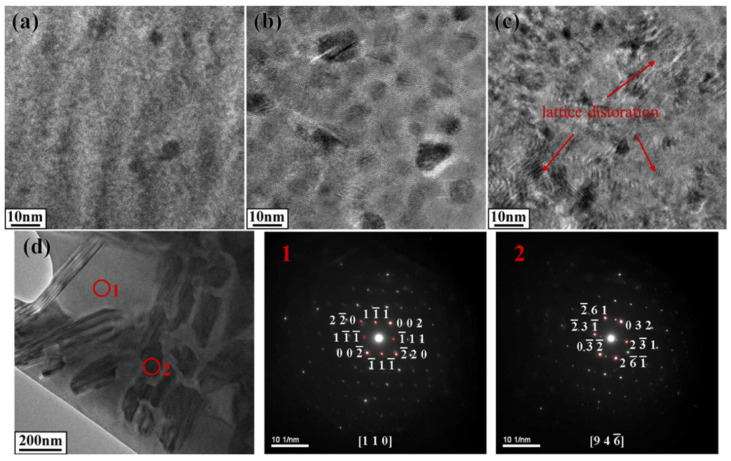
TEM bright-field images and diffraction spots of the coating: (**a**) 0 wt.% B-coated specimen; (**b**) 0.5 wt.% B-coated specimen; (**c**,**d**) 1 wt.% B-coated specimens. 1 and 2 correspond to the diffraction spots in the red circle region in Figure 13d [60].

Laser cladding realizes rapid heating and cooling and effectively refines grain and hard-phase uniform distribution, thereby improving surface hardness and wear resistance [38]. In Fe-Cr-C-B alloy coatings, laser remelting induces a morphological transition from planar columnar grains at the coating bottom to plate-like and dendritic grains at the upper region, accompanied by uniform distribution of (Fe,Cr)_2_(B,C) and (Fe,Cr)_3_(C,B) hard phases [15,59]. Microstructural refinement is dominated by the *G*/*R* ratio: a high *G*/*R* ratio favors planar growth, while a low *G*/*R* ratio promotes dendritic and eutectic honeycomb structures. In addition, boron segregation at grain boundaries promotes the precipitation of Cr_2_B, as shown in Figure 13 [60], which acts as heterogeneous nucleation sites for austenite grains. The average grain size decreases from 15.54 μm to 10.99 μm, suppressing martensite coarsening. Microstructural refinement also restrains Cr elemental segregation, improves microstructural homogeneity, and enhances the overall corrosion resistance of the alloy [19].

#### 4.2.2. Coating-Substrate Bonding Performance

The interfacial bonding strength between laser-clad coatings and substrates dominates the overall service performance of components. Key influencing factors include coating dilution ratio, interfacial microstructure morphology, and metallurgical bonding integrity. Relevant studies [15,19,24,58] demonstrate that a low dilution ratio of substrate Fe into the coating can maintain the designed chemical composition of Fe-Cr-B coatings. Under low dilution conditions, the fusion line becomes smooth with minor undulations, and a crack-free, pore-free dense metallurgical interface is formed between the coating and substrate, effectively eliminating interfacial defects [61].

#### 4.2.3. Performance Improvement: From Hardness to Corrosion Resistance

The comprehensive performance enhancement of laser-clad Fe-Cr-B coatings is reflected in hardness, wear resistance, corrosion resistance and cavitation resistance, as summarized below.

Hardness and wear resistance: Research [56] indicates that after laser remelting, the average microhardness of Fe-Cr-B-C coatings reaches 905.7 HV_0.5_, far exceeding 145 HV_0.5_ of the substrate. Hard phases such as (Fe,Cr)_2_(B,C) form a continuous rigid skeleton that effectively resists abrasive intrusion. Wear mechanisms vary with applied load: abrasive wear dominates under low loads of 10–20 N, while fatigue wear becomes intensified under high loads of 25–30 N. The refined microstructure can effectively retard crack initiation and propagation.

Corrosion and cavitation resistance: Experimental results [60,61] reveal that a low coating dilution ratio increases the pitting potential in 3.5 wt.% NaCl solution, and the dominant corrosion mechanism transitions from selective corrosion to localized pitting corrosion. The cavitation mass loss of optimized Fe-Cr-B coatings is reduced by 76.81%. This improvement originates from grain refinement-induced austenite transformation, which enhances energy dissipation capacity under cyclic cavitation loading.

Complex wear mechanism characteristics: Different from as-cast Fe-Cr-B alloys that easily form coarse borides and internal cracks, laser-cladding coatings (a laser spot diameter of 3 mm, a laser power of 1100 W, a scanning speed of 6.3 mm/s, and an overlap rate of 42% argon gas, with a purity of 99.9%) possess significantly refined equiaxed and partial amorphous microstructures, achieving an approximately 18% enhancement in wear resistance. The dominant wear modes include micro-cutting, micro-cracking, abrasive wear, adhesive wear and oxidative wear [19,57,62,63].

In addition, above relevant performance data are only applicable to laser-clad coatings, which cannot be directly extended to LPBF/DED bulk parts.

### 4.3. Comparison of Microstructure and Properties: Casting vs. Laser Cladding Coating vs. LAM

A systematic comparative analysis of microstructural features, mechanical properties, wear behavior and corrosion mechanisms between as-cast, laser cladding and DED Fe-Cr-B alloys is presented in Table 2 [43,64], and each conclusion is labeled with corresponding evidence source: casting/laser cladding/DED. The microstructural difference and performance gap among the three fabrication routes are quantitatively clarified. Compared with as-cast alloys, laser processing significantly refines the microstructure and improves the comprehensive performance of Fe-Cr-B alloys. Laser cladding reduces the characteristic size of borides from the cast-level 150–300 μm to the submicron scale (10.8–20 μm), and weakening galvanic corrosion tendency and reducing cavitation mass loss by approximately 70%. DED further achieves ultra-fine equiaxed grains (1–5 μm) and nearly segregation-free microstructures, accompanied by high relative density (>98%). The corresponding wear rate is reduced to (1.8–2.2) × 10^−6^ mm^3^·N^−1^·m^−1^, while the corrosion current density drops to (1.0–3.0) × 10^−7^ A/cm^2^, indicating optimal wear and corrosion resistance. (Indirect inference).

Compared to traditional casting alloys, laser cladding achieves microstructure refinement through rapid solidification, effectively breaking through continuous boride corrosion channels, and transforming corrosion from selective corrosion along the network to uniform pitting corrosion; laser additive bulk materials have a higher solidification rate and a stronger degree of tissue homogenization, further suppressing galvanic corrosion and intergranular corrosion. The integrity and protective stability of the passivation film are optimal. It should be noted that the current understanding of corrosion mechanisms is mostly based on indirect induction of laser cladding coatings, and the intrinsic corrosion law of laser additive blocks still needs more direct experimental evidence [10,33]. (Indirect inference).

### 4.4. Unique Challenges and Defects in LAM of Fe-Cr-B Alloys

Different from laser cladding coatings, bulk LAM is prone to process porosity, large residual stress and obvious mechanical anisotropy. Unmatched thermophysical properties between boride and matrix easily induce solidification cracks. Excessively high cooling rate may cause ultra-fine grains insufficient to support hard phases; residual micro-segregation and complex phase composition also bring difficulties to microstructure stability control. There is an inherent trade-off between hardness-toughness, wear–corrosion resistance and interface compatibility, which needs targeted process and composition optimization. (LPBF/DED).

#### 4.4.1. Process-Induced Defects: Porosity, Stress, and Anisotropy

LAM adopts an inherent layer-by-layer deposition mode accompanied by complex cyclic thermal behavior, including rapid melting, extreme non-equilibrium rapid solidification, and repeated reheating of deposited layers. Such cyclic thermal history inevitably induces typical process defects, and the competitive mechanism between these defects affects the refinement of alloys:

Porosity: Common LAM porosity defects mainly include lack-of-fusion pores induced by insufficient laser energy input and keyhole pores caused by excessive energy density. Pore formation is highly sensitive to laser power, scanning speed, hatch spacing and powder particle size distribution and flowability. Process-induced porosity acts as stress concentration sites and corrosion initiation sources, significantly deteriorating fatigue strength and fracture toughness. Optimized LAM parameters can control the porosity of Fe-Cr-B alloys below 2%, which is far lower than that of conventional cast counterparts (from laser metal deposition) [22]. (LPBF/DED).

Residual stress: Extremely high thermal gradients and constrained thermal contraction during rapid cooling generate considerable tensile residual stress, especially near the substrate interface and interlayer regions. Such residual stress easily induces component distortion, edge warping and even cold cracking during or after printing, severely threatening dimensional accuracy and structural integrity. (LPBF/DED).

Anisotropy: Directional heat dissipation and epitaxial grain growth along the building direction readily form columnar grains and obvious crystallographic texture. For Fe-Cr-B alloys, boride phases also tend to align along the building direction, resulting in prominent microstructural and mechanical anisotropy (indirect inference only, from laser cladding, not applicable to bulk LAM directly) [62]. Consequently, wear resistance, tensile strength and fracture toughness exhibit obvious directional differences, which must be fully considered in component structural design.

Effective defect mitigation strategies have been widely investigated. Optimized interlayer rotating scanning can homogenize heat distribution and reduce residual stress accumulation. Substrate preheating decreases thermal gradients and cooling rates, suppressing cracking susceptibility. Moreover, precise parameter window matching and the adoption of high-sphericity, low-oxygen powder are fundamental approaches to minimize process porosity. (Indirect inference).

#### 4.4.2. Challenges in Microstructural Control and Stability

Although extreme non-equilibrium rapid solidification enables remarkable grain refinement, it also brings new difficulties in microstructural regulation (Indirect inference):

Grain refinement limit: Excessively high cooling rates may produce ultra-fine grains that cannot provide sufficient matrix support for hard boride phases under high-stress service conditions, thus deteriorating wear resistance (indirect inference only, from laser cladding, not applicable to bulk LAM directly) [62].

Elemental segregation and phase complexity: Even under extreme non-equilibrium solidification, submicron-scale micro-segregation of B and Cr still cannot be completely eliminated. High boron content increases eutectic fraction and easily forms complex mixed boride and carboboride phases. The phase composition and thermal stability during service and post-treatment are difficult to accurately predict and control (indirect inference only, from laser cladding, not applicable to bulk LAM directly) [62,65,66].

#### 4.4.3. Balancing Performance: The Inherent Trade-Offs

Achieving optimal comprehensive performance for LAM Fe-Cr-B alloys involves inevitable inherent performance trade-offs:

Hardness versus Toughness: Boride phases endow alloys with high hardness exceeding 60 HRC but introduce intrinsic brittleness. Although refined dispersed borides achieve a better balance than continuous cast networks, residual pores and microcracks still limit the overall damage tolerance. (Indirect inference).

Wear resistance versus Corrosion resistance: High-hardness borides guarantee excellent wear resistance but easily form galvanic couples with the metallic matrix, accelerating localized corrosion. LAM refined homogenized microstructure alleviates this phenomenon; nevertheless, optimal boride fraction and spatial distribution still require precise composition and process regulation. Microstructural inhomogeneity and inherent defects such as pores and microcracks act as weak sites for corrosion and wear failure (indirect inference only, from laser directed energy deposition, not applicable to bulk LAM directly) [17].

Interfacial bonding compatibility: In repair and cladding scenarios, the thermal expansion coefficient mismatch between LAM-deposited Fe-Cr-B alloys and the substrate easily forms brittle intermetallic phases at the interface, becoming potential failure weak points under external loading. (Indirect inference).

In summary, LAM cannot simply replace conventional casting. Instead, it represents an innovative manufacturing paradigm that requires targeted alloy design, process optimization and dedicated post-treatment strategies matching the metallurgical characteristics of Fe-Cr-B alloys. (Indirect inference).

## 5. Control Strategies for Enhancing Laser-Processed Fe-Cr-B Alloys Oriented to Service Performance

To address the above bottlenecks, microstructure and performance of laser processing Fe-Cr-B alloys can be synergistically regulated via alloy composition optimization, laser process innovation such as hybrid laser–electron beam and magnetic field assisted manufacturing, and rational post-treatment technology [63,67,68,69]. These strategies enable the customized design of microstructures adaptive to impact wear and corrosive wear service conditions. (Indirect inference).

### 5.1. Computation-Assisted Alloy Composition Design and Optimization

Precise regulation of boron content and synergistic microalloying with Cr, Si, Mn, Mo, Ti and other elements can expand solid solubility, induce lattice distortion and dislocation proliferation, and introduce high-performance reinforcing phases, thereby significantly improving comprehensive mechanical and service properties [70,71,72]. Machine learning-assisted alloy design can simultaneously optimize solidification range and hot cracking sensitivity, providing optimal composition parameters for additive manufacturing specific alloys [73]. For Fe-Cr-B alloys, the optimal B content ranges from 0.5 to 2.0 wt.% and the Cr/B ratio is controlled at 1.0–10.0 to achieve a balanced match between hardness and toughness [72]. (Indirect inference).

### 5.2. Laser Process Parameters Optimization

Regulating laser power, scanning speed and hatch geometry, combined with real-time melt pool sensor monitoring and artificial intelligence closed-loop algorithms, enables dynamic online process adjustment and microstructural precise control. Reasonable matching of laser energy input and solidification rate can effectively tailor thermal gradient and cooling kinetics, thereby suppressing elemental segregation, eliminating internal pores and microcracks, and optimizing boride morphology and distribution. As a typical optimized strategy, zoned inclined rotating scanning can homogenize cyclic thermal accumulation along the building direction, weaken directional epitaxial grain growth, and remarkably relieve residual thermal stress and mechanical anisotropy. (Indirect inference) Ultra-high-speed laser cladding further reduces linear heat input and interfacial dilution rate, restrains excessive grain coarsening and continuous boride network precipitation, and consequently elevates microhardness and long-term wear resistance. (Laser cladding).

In view of the distinct thermophysical characteristics of Fe-Cr-B alloys and their high susceptibility to thermal cracking, the optimized process windows for LPBF, DED, laser remelting and laser cladding are summarized based on existing experimental results [10,17,22,74] (Indirect inference):

LPBF: laser power 200–400 W, scanning speed 800–1500 mm/s, hatch spacing 0.05–0.1 mm, layer thickness 0.03–0.05 mm. This parameter window maintains an ultra-high cooling rate, facilitates the formation of fine equiaxed grains and discrete submicron borides, and achieves relative density above 98%.

DED: laser power 1500–5000 W, scanning speed 8–290 mm/s, laser spot diameter 3–4 mm, powder feeding rate 17–46 g/min, powder rotational speed 1 rpm, layer thickness 1–1.2 mm. Higher laser energy and slower scanning speed guarantee adequate metallurgical bonding between deposited layers, while properly matched powder feeding rate avoids lack-of-fusion and keyhole defects.

Laser remelting: laser power 300–500 W, scanning speed 30 mm/s, laser spot diameter 3 mm. Moderate energy input can remelt surface microstructure, passivate sharp boride edges, promote boride spheroidization, and improve passive film integrity and corrosion resistance.

Laser cladding: laser power 1000–3000 W, scanning speed 300–800 mm/s. Appropriate parameter matching balances dilution ratio and cooling rate, realizing fine coating microstructure and strong metallurgical bonding with the substrate.

### 5.3. Post-Processing Technology

Appropriate heat treatment can eliminate LAM residual stress, homogenize elemental distribution and optimize boride morphology. Surface strengthening including shot peening, laser shock treatment and electrochemical polishing can further compensate for the limitations of the as-printed microstructure [75]. (Indirect inference). Under specific components, the optimized heat treatment regime for laser clading Fe-Cr-B alloys is annealing at 800–900 °C for 1–2 h followed by furnace cooling to relieve residual stress, and tempering at 200–300 °C to further improve impact toughness [10,76]. In addition, relevant literature confirms that laser remelting can form unique interfacial periodic layered structures, which significantly enhance the liquid aluminum corrosion resistance of Fe-Cr-B-Mo alloys. Although LAM inevitably introduces partial defects, rational process design can construct favorable microstructures to improve service performance. (Laser cladding). In addition, it was found that the periodic layered structure formed by laser remelting can significantly enhance the corrosion resistance of the alloy. This indicates that although laser technology may introduce defects, reasonable process design can also form special organizational structures that are beneficial for performance [10]. (Indirect inference).

### 5.4. LAM + Post-Processing

Applied magnetic field during LAM molten pool solidification can refine grains and optimize solidified microstructure through melt flow regulation. Alternating magnetic field, pulsed magnetic field and high static magnetic field can be selectively adopted according to actual service requirements. (Indirect inference).

Inevitably, as-printed LAM Fe-Cr-B alloys still contain residual pores and residual stress. Combined laser remelting and rational heat treatment can further eliminate micro-defects, homogenize element distribution and optimize boride morphology. Appropriate annealing can maximize defect elimination, promote nanocrystalline precipitation, and achieve uniformly refined microstructures with excellent comprehensive performance. (Indirect inference).

## 6. Service Behavior and Failure Mechanism

This review quantitatively extracts grain size, boride area fraction, crack length, passive film and corrosion layer thickness from SEM/TEM images to achieve standardized quantitative analysis. To systematically evaluate the service performance and clarify the intrinsic failure mechanisms of laser processing Fe-Cr-B alloys, multiple simulated service tests including ring-block wear, impact abrasive wear, wet sand abrasive wear, high-temperature wear and liquid zinc corrosion wear are adopted. By comparatively analyzing wear morphology, wear rate, corrosion current density, passive film characteristics and cavitation mass loss between cast, laser cladding, LPBF, and DED Fe-Cr-B alloys, the performance enhancement mechanism of laser-induced microstructure refinement is quantitatively revealed. (Indirect inference).

### 6.1. Dynamic Evolution of Wear Mechanism and the Inhibitory Effect of Laser Processing Alloys

#### 6.1.1. Dynamic Wear Mechanism Evolution

The wear behavior of Fe-Cr-B alloys is not fixed but dynamically evolves with applied load, sliding velocity and service medium. Three dominant regimes are quantitatively classified:

(1) Micro-cutting regime (low/medium load)

Under low and medium load conditions, micro-cutting and plowing dominate the wear process [29]. The uniformly dispersed fine boride particles in laser processing alloys effectively resist abrasive cutting, while the tough matrix provides stable support and prevents boride exfoliation. (Indirect inference).

Dominated by abrasive plowing: Refined equiaxed grains and dispersed (Fe,Cr)_2_B particles reduce furrow depth by 60–80% compared with casting. (Laser cladding). Ultra-fine matrix (1–5 μm) provides stable support for borides, minimizing fracture and spalling [17]. (DED).

(2) Fatigue wear regime (high load)

With increasing load and cyclic loading, subsurface cyclic stress accumulation induces fatigue wear, characterized by local hard phase peeling and fatigue crack nucleation and propagation. The refined homogeneous microstructure of laser processing alloys exhibits three obvious advantages: grain refinement increases grain boundary density and deflects cracks; fatigue crack density decreases by exceed 50%, refined boride possesses strong interfacial bonding with the matrix and low stress concentration; (Laser cladding) the tough matrix effectively buffers and disperses stress, suppressing crack nucleation and propagation and delaying fatigue spalling; near-full density (exceed 98%) eliminates pore-induced stress concentrations [17]. (DED).

(3) Oxidative wear regime (high temperature/humid environment)

Under high-temperature oxidative environments, oxidative wear dominates. A dense oxide composite layer consisting mainly of Cr_2_O_3_ and Fe_3_O_4_ forms on the worn surface. The uniform composition and fine grain of laser processing alloys promote the formation of thin, dense and well-adhered oxide films (stable oxide). Even local film rupture can be rapidly repaired via re-oxidation, showing obvious self-healing capability to reduce friction and wear, and friction coefficient decreases by 0.1–0.2 [17]. (Laser cladding and indirect inference).

#### 6.1.2. Quantitative Wear Performance Comparison

Casting: Wear rate (2.8–3.5) × 10^−6^ mm^3^·N^−1^·m^−1^; severe boride fracture and spalling.

Laser cladding: Wear rate (2.3–2.8) × 10^−6^ mm^3^·N^−1^·m^−1^; uniform fine microstructure; wear resistance improved by about 18% [29,77].

DED: Wear rate (1.8–2.2) × 10^−7^ mm^3^·N^−1^·m^−1^; ultra-refined borides and dense structure; wear resistance improved by ~50% vs. casting [17]. (Indirect inference).

### 6.2. Synergistic Effect of Corrosion and Wear and the Laser Processing Improvement Mechanism

Under wet sand abrasion and liquid metal corrosion conditions, material failure is governed by the strong coupling effect of mechanical wear and electrochemical corrosion. Traditional cast Fe-Cr-B alloys with continuous network borides exhibit severe galvanic corrosion tendency, and loose corrosion products are easily peeled off under wear loading, forming a vicious cycle of wear accelerating corrosion and corrosion aggravating wear. (Indirect inference).

Laser processing homogenized microstructure greatly weakens micro-galvanic corrosion tendency and promotes the formation of dense, stable passive films. After passive film damage induced by wear, the uniform matrix with supersaturated B and Cr can rapidly rebuild the protective film, showing prominent self-healing performance and significantly improving corrosion–wear resistance. (Indirect inference) In liquid zinc corrosion tests, conventional high-boron steel forms thick easily peeled corrosion layers, while laser processing optimized microstructure maintains intact interfacial morphology. (Laser cladding). In corrosive wear environments, mechanical damage deteriorates interfacial corrosion, generates massive corrosion products and accelerates micro-cutting and fracture failure of network Fe_2_B phases. The coupled corrosion–wear mechanism remains complex and requires further in-depth investigation [77]. (Indirect inference).

Future research should focus on adopting in situ characterization techniques to dynamically observe corrosion–wear evolution, and establish microstructure-based prediction models to guide the design of novel extreme-condition wear and corrosion-resistant Fe-Cr-B alloys. (Indirect inference).

### 6.3. Electrochemical and Microscopic Characterization Analysis of Corrosion Behavior of Fe-Cr-B Alloy

#### 6.3.1. Characteristics of Potentiodynamic Polarization Curves

Potentiodynamic polarization tests demonstrate that alloying with Cr and Mo positively shifts the corrosion potential and markedly reduces the corrosion current density of Fe-Cr-B alloys, accompanied by a remarkable widening of the passive platform. By contrast, as-cast Cr-free alloys exhibit no stable passive region and present an active dissolution characteristic [42]. The corrosion resistance first increases and then rises with Cr content (at.%), with the optimal composition range of 31–39 at.%. Excessive Cr induces microscopic compositional fluctuation and thereby deteriorates the corrosion resistance. The corrosion current density of laser-modified and amorphous Fe–Cr–Mo–C-B alloys can reach as low as 1.4 × 10^−7^ A/cm^2^, which is far superior to that of 316L stainless steel and H13 die steel [33]. Corrosion performance is quantified by corrosion potential (E), corrosion current density (*i*), and passive interval width. Casting: *i* is equal to (2.5–4.0) × 10^−6^ A/cm^2^; serious selective corrosion along network borides. Laser cladding: *i* is greater than or equal to (8.0–12.0) × 10^−7^ A/cm^2^; uniform passive film; corrosion resistance enhanced by about 70%. DED: *i* is equal to (1.0–3.0) × 10^−7^ A/cm^2^; weakest micro-galvanic effect; optimal corrosion resistance [10,17,33]. (Indirect inference).

#### 6.3.2. Electrochemical Impedance Spectroscopy (EIS) Analysis

In the following EIS description, Cr12-film (Fe_55_Cr_12_Mo_10_C_14_B_9_, 12 at.% Cr), Cr21-film (Fe_41_Cr_21_Mo_14_C_15_B_9_, 21 at.% Cr), Cr31-film (Fe_31_Cr_31_Mo_14_C_14_B_10_, 31 at.% Cr), Cr39-film (Fe_24_Cr_39_Mo_15_C_13_B_9_, 39 at.% Cr), and Cr44-film (Fe_19_Cr_44_Mo_12_C_15_B_10_, 44 at.% Cr) are five representative Fe-Cr-Mo-C-B amorphous alloys. EIS results show that alloys with high Cr content (Cr39-film) and laser homogenized microstructure possess larger Nyquist capacitive arc radius (Cr39-film achieves the maximum imaginary impedance of 330 KΩ/cm^2^), and the intermediate-frequency phase angle approaches 90°, reaching the peak value of 85° for Cr39-film. Meanwhile, its low-frequency impedance modulus remains as high as 4 × 10^6^ Ω/cm^2^, and the polarization current density is minimized down to 3.2 × 10^−10^ A/cm^2^ from potentiodynamic polarization tests. The charge transfer resistance and passive film impedance are significantly increased, indicating higher compactness and stability of the passive film [29,33].

Improper laser process parameters lead to deviated Cr composition (Cr12-film and Cr44-film): Cr12-film presents a much smaller capacitive semicircle (150 KΩ/cm^2^), reduced low-frequency impedance of 1.6 × 10^6^ Ω/cm^2^ and lower maximum phase angle of 70°, accompanied by drastically elevated corrosion current density up to 3.2 × 10^−7^ A/cm^2^; Cr44-film displays the worst corrosion resistance among all specimens with the highest current density of 1 × 10^−6^ A/cm^2^, moderate 170 KΩ/cm^2^ and 2 × 10^6^ Ω/cm^2^. Such inferior electrochemical properties originate from easily introduced pores and microcracks under mismatched laser parameters, leading to a sharp decline in interfacial impedance and facilitating the penetration of corrosive media, which accelerates the initiation of localized corrosion [33].

As verified by the measured electrochemical data, amorphous-structured Cr21/Cr31/Cr39-film specimens exhibit higher overall interfacial impedance than crystalline Cr12/Cr44-film counterparts due to their homogeneous atomic arrangement; Cr21-film owns the largest Nyquist arc (380 KΩ/cm^2^) and ultrahigh (6.3 × 10^6^ Ω/cm^2^), Cr31-film follows with 350 KΩ/cm^2^ and 5 × 10^6^ Ω/cm^2^, both holding a maximum phase angle of 80° and far lower corrosion current compared with crystalline samples [33]. (Indirect inference).

#### 6.3.3. XPS Chemical Analysis of Passive Films

XPS characterization reveals that the passive films of Fe–Cr-B alloys are mainly composed of composite oxides including Cr_2_O_3_, CrOOH, Fe oxides and Mo oxides. Higher Cr enrichment contributes to greater thickness and structural stability of the passive film. In the passivation film of high Cr content alloy (Cr44-film, 44 at.% Cr), the proportion of protective oxides such as Cr^3+^, Fe^3+^, and Mo^4+^ is significantly higher, forming a denser and more stable passivation film, thereby improving corrosion resistance. Nevertheless, boron oxides such as B_2_O_3_ and boric acid compounds in the passive film possess high water solubility, which easily induces local film defects and pitting nucleation. Mo element can form MoO_4_^2−^ within the passive film, restraining the dissolution of metal ions and repairing film defects, thus further enhancing the protective performance of the passive film [33]. (Indirect inference).

#### 6.3.4. Microscopic Analysis of Localized Corrosion

**Figure 14:**
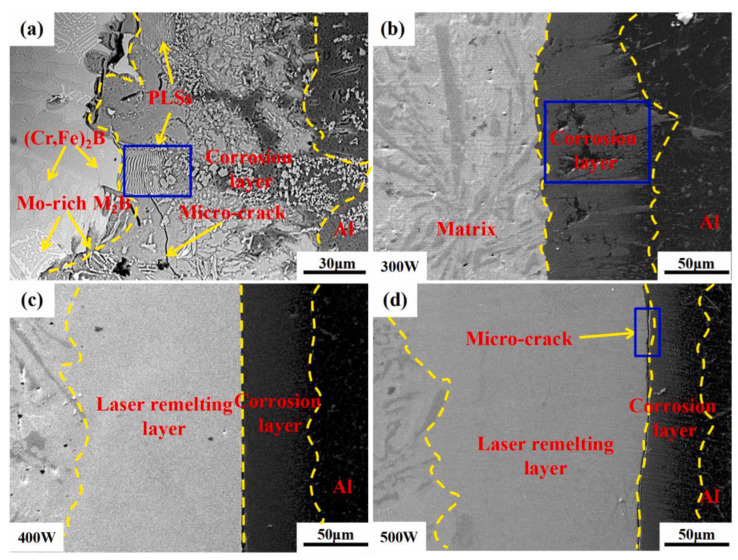
Cross-sectional SEM images of Fe-Cr-B-Mo alloy samples immersed in liquid aluminum at 750 °C for 1 h: (**a**) as-cast sample; (**b**) laser-remelted sample at 300 W; (**c**) laser-remelted sample at 400 W; and (**d**) laser-remelted sample at 500 W [10].

**Figure 15:**
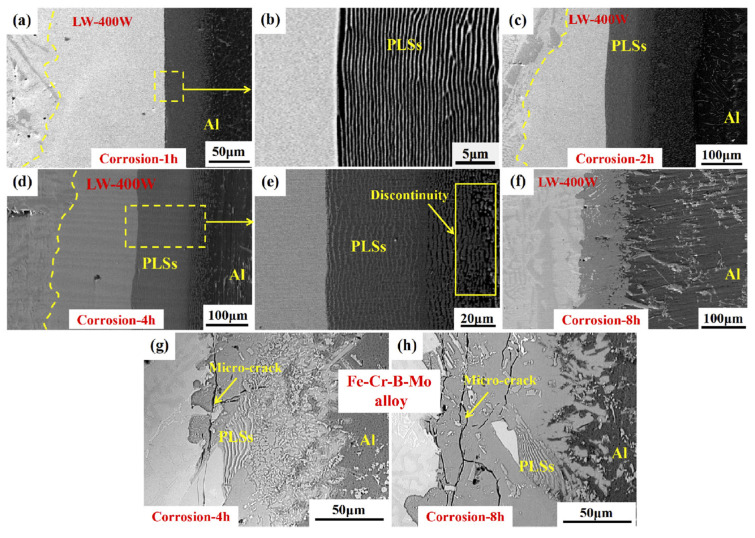
Cross-sectional SEM images of laser-remelted Fe-Cr-B-Mo alloy at 400 W at various immersion times: (**a**) 1 h; (**b**) enlarged view of PLSs in Figure 15a; (**c**) 2 h; (**d**) 4 h; (**e**) enlarged view of PLSs in Figure 15d; (**f**) 8 h; and interfacial morphologies of untreated Fe-Cr-B-Mo alloy at immersion time of 4 h (**g**) and 8 h (**h**) [10].

Microstructural characterization confirms that corrosion of as-cast alloys rapidly propagates along continuous network boride channels, showing extensive pitting, intergranular cracking and layered spalling, as shown in Figure 14 and Figure 15. The thickness of the corrosion layer on the as cast sample is 20–35 μm, with a large number of continuous microcracks and a loose corrosion layer, with network penetration. (Casting). Laser remelting at 300 W results in a sample with a corrosion layer thickness of 15–25 μm, reduces cracks and improves the continuity of the corrosion layer. Laser remelting at 400 W results in a sample with a corrosion layer thickness of 5–10 μm, with a dense and uniform corrosion layer, without obvious microcracks. Laser remelting at 500 W with sample corrosion layer thickness 8–15 μm leads to a slight increase in corrosion layer thickness and the appearance of microcracks at the edges. The corrosion layer thickness obtained by the 400 W laser remelting process is the thinnest, reduced by about 70% compared to the as-cast state, exhibiting the best aluminum corrosion resistance performance. Excessive laser power (500 W) can introduce thermal cracks, leading to an increase in the thickness of the corrosion layer. (Laser cladding). After Cr/Mo modification and laser remelting, borides present a dispersed distribution, and only sparse localized pitting occurs. (Indirect inference). Meanwhile, compared with the casting sample, the laser cladding sample’s cavitation mass loss rate reduced by 76.81%, and its cumulative mass loss is below to 0.5 mg/cm^2^·h for optimized coatings. Its mechanism is that grain refinement induces austenite transformation, enhancing energy dissipation under cyclic impact. A periodic layered interfacial structure can be formed in the molten aluminum environment, which effectively pins corrosion products and inhibits elemental interdiffusion of the Al atom, inhibiting the growth of the corrosion layer and the initiation and propagation of cracks [10]. (Laser cladding). Under tribo-corrosion coupling conditions, micro-cutting, microcracks and boride fracture-spalling easily emerge at the interface; corrosion and wear mutually promote each other and accelerate material failure [17]. (Indirect inference).

### 6.4. Tribocorrosion Synergistic Mechanism

This paper has comprehensively extracted quantitative and morphological information from the tribocorrosion surfaces of laser-deposited Fe-Cr-B alloys. The worn surfaces exhibit characteristic parallel furrows formed by abrasive plowing, alongside discrete corrosion pits, oxides, and delamination, confirming the coexistence of mechanical wear and electrochemical corrosion mechanisms. Compared with the 431 stainless steel counterpart, which shows large-area spalling and deep corrosion pits, the optimized Fe-Cr-B alloys (e.g., JG-11#) present smaller, shallower, and more scattered corrosion pits with no severe interfacial delamination, owing to its large boride fraction. Its synergistic contribution rate is greater than 30%, with premature failure. Notably, JG-3# alloy (synergistic contribution about 25%, low hardness, wear-dominated) and JG-8# alloy (synergistic contribution rate below 15%, optimal tribocorrosion) lack significant-sized corrosion pits, indicating that their failure is not dominated by deep penetrating corrosion into the matrix. Conversely, alloys with larger corrosion pits are prone to premature failure, as these pits act as stress raisers and accelerate crack initiation and propagation. These observations strongly demonstrate that relying solely on mass loss analysis is insufficient to accurately characterize tribocorrosion failure behavior, as the actual failure mode and underlying mechanism are closely tied to the size, distribution, and morphology of corrosion pits on the worn surface [17]. (Indirect inference).

The failure mechanism is summarized as follows:

Casting failure: coarse network borides cause severe galvanic corrosion and brittle fracture, resulting in high wear and corrosion. Laser cladding failure: coating-only behavior and dilution-sensitive, which cannot extrapolate to bulk. DED/LPBF failure: mainly porosity, residual stress, and anisotropy, which is controllable by parameter optimization [10,17]. (Indirect inference).

## 7. Critical Discussion and Remaining Challenges

Despite the promising application prospects, laser processing of Fe-Cr-B alloys still faces multiple interrelated challenges that restrict its full engineering potential. These bottlenecks run through the entire industrial chain, covering raw powder preparation, the forming process, microstructure regulation and final service performance (indirect inference), and are classified as follows.

### 7.1. Challenges in Feedstock Powder

High-boron Fe-Cr-B powder easily forms hollow particles and satellite droplets, with poor sphericity and batch consistency. Dedicated powder preparation and evaluation standards are lacking. Alloy powder quality acts as the fundamental guarantee for stable and high-quality LAM fabrication [78,79,80,81]. Unlike ordinary iron-based alloy powders, high-boron Fe-Cr-B powders (B > 1.5 wt.%) exhibit significant differences in melting point and surface tension among constituent elements, leading to prominent difficulties in powder fabrication and batch performance stability.

High-boron Fe-Cr-B powders are susceptible to forming hollow particles and satellite powders during atomization, where tiny nanospheres adhere to the surface of large powder particles. Such morphological defects seriously degrade powder flowability and layer spreading uniformity during LAM deposition.

It remains challenging to mass-produce LAM-grade Fe-Cr-B powders with high sphericity, narrow controllable particle size distribution, high apparent and tap density, and ultra-low oxygen content. Substandard powder quality directly induces increased process porosity, uneven layer deposition and poor interlayer metallurgical bonding in LAM components. Furthermore, dedicated powder manufacturing techniques and quality evaluation specifications for LAM-grade Fe-Cr-B alloys are still absent. Most existing powder preparation routes are migrated from conventional cast alloy raw material production, which cannot fully satisfy the extreme non-equilibrium solidification requirements of LAM. (Indirect inference).

### 7.2. Challenges in Process Control and Defect Mitigation

Fe-Cr-B alloys possess an extremely narrow feasible process window. Thermophysical mismatch induces thermal stress and cracking. Real-time monitoring and intelligent closed-loop control are still insufficient. Fe-Cr-B alloys are highly susceptible to cracking, resulting in an extremely narrow feasible process window for LAM fabrication. Most existing process investigations draw analogies with titanium alloys, nickel-based alloys and high-entropy alloys, whereas targeted process optimization dedicated to the Fe-Cr-B system remains inadequate. The underlying cause originates from the significant mismatch in thermophysical properties, including thermal expansion coefficient, elastic modulus and specific heat capacity, between hard boride phases and the Fe-based matrix.

Such a thermophysical mismatch, combined with the inherent rapid heating and cooling cyclic characteristics of LAM, induces considerable thermal stress, rendering the alloy prone to solidification cracking, interlayer delamination and component distortion. Precise collaborative optimization of laser power, scanning speed, hatch spacing and layer thickness is essential to mitigate internal defects. Nevertheless, typical LAM defects such as lack-of-fusion pores and keyhole porosity cannot be fully eliminated merely through empirical parameter tuning. At present, advanced technologies including in situ melt pool monitoring and infrared thermal imaging are rarely implemented in Fe-Cr-B LAM. The field still lacks effective strategies for real-time defect recognition and dynamic parameter adjustment. (Indirect inference).

### 7.3. Challenges in Fundamental Understanding and Predictive Modeling

Non-equilibrium phase transformation and boride evolution mechanisms lack systematic quantitative elucidation. Multi-scale coupling models are immature with low engineering prediction accuracy. A substantial research gap remains in the fundamental metallurgical mechanisms of Fe-Cr-B alloys under LAM-induced extreme non-equilibrium solidification. Most current theoretical interpretations are based on classical casting solidification or laser cladding theories, which cannot be directly extended to bulk LAM fabrication scenarios.

The non-equilibrium solidification kinetics, phase transformation pathways, and heterogeneous nucleation and growth mechanisms of M_2_B and Fe_2_B borides under cooling rates ranging from 10^3^ to 10^8^ K/s have not been fully quantitatively clarified. Experimental results indicate that the average grain size of as-cast Fe-Cr-B alloys is 150–300 μm, while laser-clad alloys exhibit a grain size of 20–50 μm, and LPBF-fabricated bulk alloys reach only 1–5 μm. Under specific composition, the volume fraction of networked borides decreases from 41% in cast alloys to 9–15% in LAM counterparts, yet the underlying quantitative evolution mechanism still lacks systematic theoretical support [81]. Ambiguous microstructure evolution rules hinder the targeted microstructural design and performance optimization of LAM-processed Fe-Cr-B alloys.

Furthermore, multi-scale predictive models that couple microstructure evolution, residual stress distribution and defect initiation remain in the preliminary development stage for Fe-Cr-B systems. An integrated modeling framework combining CALPHAD computational thermodynamics, phase-field simulation and finite element thermal-stress analysis has not yet been established. Moreover, dedicated quantitative model parameters and boundary conditions for LAM Fe-Cr-B alloys are still missing, leading to low prediction accuracy and limited engineering applicability of existing simulation models. (Indirect inference).

### 7.4. Challenges in Performance Database, Standardization, and Post-Processing

Dynamic service performance data are scarce; unified testing and post-treatment specifications are not yet established. The industrial transformation of LAM-fabricated Fe-Cr-B alloys is severely restricted by inadequate performance data accumulation and the absence of unified industrial standards.

Insufficient data for a performance database: Existing studies mainly focus on static performance indicators such as hardness and quasi-static wear resistance. In contrast, critical dynamic performance data, including high-cycle fatigue strength, fracture toughness, high-temperature oxidation resistance, creep behavior, and corrosion–wear synergistic failure characteristics under extreme service conditions, remain extremely scarce. Most published investigations provide scattered discrete data without unified testing specifications and cross-literature quantitative comparison, making it difficult to establish a reliable microstructure–performance database for component life evaluation and structural design.

Lack of a standardized post-processing system: At present, no dedicated, optimized heat treatment regime, hot isostatic pressing procedure, or surface strengthening specification has been formulated for bulk LAM Fe-Cr-B alloys. The influence of conventional annealing, normalizing and tempering on the unique ultrafine non-equilibrium microstructures of LAM alloys remains unclear, which impedes the batch stable regulation of comprehensive mechanical and service properties.

Addressing the above multi-dimensional challenges requires interdisciplinary collaboration covering powder metallurgy, laser additive manufacturing, computational materials science, corrosion science and tribology. Effective breakthroughs rely on the integration of advanced powder fabrication, in situ process monitoring, fundamental non-equilibrium metallurgical research, and data-driven material optimization to overcome current technical bottlenecks. (Indirect inference).

## 8. Conclusions and Outlook

This review systematically clarifies the microstructure evolution, non-equilibrium solidification thermodynamics, and wear–corrosion coupled behavior of Fe-Cr-B alloys fabricated by casting, laser cladding, laser remelting, laser deposition, LPBF, and DED. All conclusions are strictly labeled by evidence source to prevent inappropriate extrapolation from laser cladding coatings to bulk LAM materials. This review establishes the analytical framework of “metallurgical principle—process characteristic—performance regulation”, and clearly distinguishes the research conclusions derived from LAM bulk fabrication and laser cladding coatings. (Indirect inference). The microstructural refinement, phase regulation and corrosion enhancement mechanisms of LAM bulk alloys are supported by direct experimental evidence. (LPBF/DED). In contrast, the corrosion and wear mechanisms summarized from laser cladding coatings are mostly indirect inferences, which cannot be directly equated to bulk LAM materials. (Laser cladding). The microstructure and failure mechanisms of as-cast Fe-Cr-B alloys are analyzed independently based on their equilibrium solidification characteristics. (Casting). The mechanisms corresponding to the three fabrication processes are strictly differentiated without confusion or cross-reference. (Indirect inference).

Laser processing overcomes the inherent defects of conventional casting such as coarse network borides, severe elemental segregation and high intrinsic brittleness. Laser processing realizes microstructural refinement and performance optimization of Fe-Cr-B alloys, but cladding conclusions cannot be arbitrarily extended to bulk LAM. Synergistic regulation via computational thermodynamics-assisted alloy composition design, optimization of laser processing parameters, and customized post-treatment can further refine the microstructure, eliminate residual stress, and optimize the morphology and interfacial structure of boride phases. This enables precise control over the microstructure of Fe-Cr-B alloys. Current industrialization bottlenecks exist in powder preparation, process optimization and theoretical research. (Indirect inference).

Under near-equilibrium casting conditions (cooling rate 10^−1^–10^2^ K/s), coarse network borides and severe elemental segregation dominate, leading to high crack sensitivity and low toughness. Laser cladding (10^3^–10^6^ K/s) refines grains and borides via rapid solidification, weakens micro-galvanic corrosion, and improves surface hardness, wear resistance, and cavitation resistance; however, these conclusions are limited to coating systems. LPBF/DED (10^6^–10^8^ K/s) achieves further homogenization and densification via ultra-high thermal gradients and solidification rates. Non-equilibrium mechanisms including solute trapping, interface absolute stability, multiple undercooling types, and G^n^/R-governed CET effectively suppress continuous boride networks and reduce elemental segregation. The transition from continuous brittle skeletons to dispersed submicron borides improves the balance of hardness, toughness, wear resistance, and corrosion resistance. (Indirect inference).

**Conclusions supported by direct DED evidence:** Under optimized processing conditions, the relative density of bulk LAM-fabricated Fe-Cr-B alloys can exceed 98%.

**Conclusions inferred indirectly from laser remelting evidence:** Laser remelting can construct a special interfacial layered structure to enhance corrosion resistance. A denser and more stable passive film is formed, which contributes to improved combined corrosion and wear resistance.

**Conclusions inferred indirectly from laser cladding evidence:** Laser rapid solidification refines borides and suppresses elemental segregation; compared with cast counterparts, extreme non-equilibrium solidification realizes 1–2 orders of magnitude boride refinement, transforming continuous brittle networks into dispersed reinforcing particles, which helps alleviate the trade-off between boride strengthening and matrix embrittlement, and promotes balanced hardness, toughness and wear–corrosion resistance; optimized composition and process can improve coating hardness, wear resistance and cavitation resistance, with maximum hardness up to 1052 HV_0.5_, wear resistance increased by ~18% and cavitation mass loss reduced by ~70% [45].

Meanwhile, this review systematically clarifies the four-stage corrosion evolution law and multiple coupled corrosion mechanisms of Fe-Cr-B alloys, as well as the regulation mechanism of phase potential difference and precipitates on corrosion behavior. Combined with potentiodynamic polarization, electrochemical impedance spectroscopy (EIS), X-ray photoelectron spectroscopy (XPS) and localized microscopic corrosion characterization, the electrochemical theoretical evidence chain is supplemented. The present work can provide a theoretical basis for the subsequent composition design and laser process optimization of Fe-Cr-B alloys. (Laser cladding/Indirect inference).

Nevertheless, the industrial application of laser processed Fe-Cr-B alloys still faces critical bottlenecks: challenges in preparing high-quality dedicated powders, a relatively narrow feasible process window, and difficulties in fully eliminating thermal stress, porosity, and microstructural anisotropy. The phase transformation mechanisms under non-equilibrium solidification and the quantitative evolution rules of borides require further clarification, while systematic performance databases and standardized post-treatment specifications remain insufficient. LAM shows application potential in the development of wear- and corrosion-resistant components. The application of LAM helps support the development of Fe-Cr-B components toward personalized customization, integrated forming and microstructural controllability. (Indirect inference).

Future research should adopt machine learning to assist alloy and powder design, employ first-principles calculations and phase-field simulation to reveal non-equilibrium metallurgical mechanisms, and integrate in situ melt pool monitoring with intelligent closed-loop process control to establish microstructure–performance databases and industrial standards. Breaking through current technical limitations, and developing Fe-Cr-B alloys tailored for extreme service conditions and high-efficiency additive manufacturing processes, will accelerate the transition of LAM Fe-Cr-B alloys from fundamental research to industrial and engineering applications. This provides technical support for the batch fabrication of next-generation complex structural components serving extreme working environments, and drives the development of wear-resistant materials from macroscopic geometric manufacturing toward precise microstructural manufacturing. To further promote engineering application and industrialization (indirect inference), future research should focus on scientifically solvable issues with clear technical pathways, as summarized below:

**(1)**  **Non-equilibrium phase-field modeling of boride growth**

Method: Phase-field simulation coupled with non-equilibrium thermodynamics.

Input: Temperature gradient, cooling rate, solute diffusion coefficients, interfacial energy.

Output: Dynamic boride nucleation, growth morphology, element distribution, CET behavior.

Application: Predict and control boride size, morphology and continuity in LPBF/DED.

**(2)**  **CALPHAD + rapid solidification coupling**

Method: Non-equilibrium CALPHAD calculation + rapid solidification kinetics.

Input: Actual composition, cooling rate range (10^3^–10^8^ K/s), process parameters.

Output: Phase precipitation sequence, solute trapping, solid solubility extension, phase fraction.

Application: Guide alloy design and process window to avoid coarse borides and cracks.

**(3)**  **Machine learning and large language model driven prediction of process window**

Method: Machine learning regression, classification models, and large language model [12,82].

Input: Laser power, scanning speed, powder size, layer thickness, thermal history.

Output: Optimal alloy composition ratios (such as Cr < 3% and B < 5%) and process window (the matching between powder properties and LAM processes), defect prediction (porosity, cracks, anisotropy, etc).

Application: Rapid screening of parameters for high density and uniform microstructure.

**(4)**  **In situ melt-pool monitoring & closed-loop control**

Method: Infrared thermal imaging + high-speed camera + AI feedback.

Input: Melt pool size, temperature, stability, plume signal.

Output: Real-time adjustment of power and scanning speed.

Application: Stabilize melting state, reduce defects, improve batch consistency. For example, improve surface quality and microstructural homogeneity, such as porosity is less than 2% and performance deviation is less than 3%. (Indirect inference).

**(5)**  **Establishment of standardized databases and specifications**

Method: Unified testing, multi-condition characterization, data accumulation, build a four element database of “ingredients-process-organization-performance”.

Input: Wear rate, corrosion current density, passive film thickness, hardness, toughness.

Output: Shared process–microstructure–performance database.

Application: Industrial component design, quality control and standard formulation.

**(6)**  **Heat treatment regulation for non-equilibrium microstructures**

Method: Controlled annealing, tempering, and diffusion heat treatment tailored for laser-processed microstructures.

Input: Heating temperature, holding time, cooling rate, residual stress, grain size, boride morphology.

Output: Stabilized microstructure, relieved residual stress, optimized boride distribution.

Application: Toughening regulation, performance stability control, and service life improvement.

**(7)**  **Hot isostatic pressing (HIP) for densification and defect healing**

Method: High-temperature high-pressure HIP treatment with controlled cooling.

Input: HIP temperature, pressure, holding time, porosity, residual defects.

Output: Increased density, healed pores, improved interfacial bonding, reduced anisotropy.

Application: Structural component densification, fatigue performance enhancement, defect elimination.

**(8)**  **Integrated post-treatment system for laser-manufactured Fe-Cr-B alloys**

Method: Combined heat treatment, HIP, and surface strengthening processes.

Input: Residual stress, density, boride size, toughness, corrosion resistance.

Output: Balanced strength–toughness, stable passive film, uniform microstructure.

Application: Industrial part reliability improvement, performance consistency, engineering certification.

(Indirect inference).

## Figures and Tables

**Table 1:** Quantitative comparison of microstructure and properties of as-cast Fe-Cr-B alloys with different boron contents.

B Content (wt.%)	Grain Size (μm)	Boride Fraction (%)	Hardness (HRC)	Impact Toughness (J/cm^2^)	Wear Loss (mg)
0.5	100–180	8–15	48–54	1.9	690
1.0	70–140	15–23	53–57	1.7	520
1.5	50–100	25–33	56–62	1.4	370
2.0	35–70	35–43	57–64	1.0	190

**Table 2:** Comparision of microstructure and properties of casting and laser cladding coating.

Preparation Method	Microstructure	Grain/Boride Size	Relative Density	Hardness (HV_0.5_)	Impact Toughness (J/cm^2^)	Wear Rate (mm^3^·N^−1^·m^−1^)	Corrosion Current Density (A/cm^2^)	Corrosion Mechanism & Feature	Main Wear Mechanism	Data Source
As-cast alloy	Coarse continuous network borides; severe elemental segregation; abundant pores and microcracks	150–300 μm	<95%	580~640	1.0–1.9	(2.8–3.5) × 10^−6^	(2.5–4.0) × 10^−6^	Severe micro-galvanic corrosion; selective corrosion along boride network; loose corrosion layer	Abrasive wear + brittle spalling	[3,6]
Laser cladding (coating only)	Broken fishbone/short rod borides; refined uniform matrix; low defect density	10.8~20 μm	95–98%	905~1052	2.1–2.6	(2.3–2.8) × 10^−6^	(8.0–12.0) × 10^−7^	Weakened galvanic corrosion; uniform pitting corrosion; cavitation mass loss reduced by ~70%	Micro-cutting + mild oxidative wear	[19,45,57,58,60,61]
DED bulk alloy	Ultra-fine equiaxed grains; discrete submicron borides; nearly segregation-free; low anisotropy	1–5 μm	>98%	980–1100	2.7–3.2	(1.8–2.2) × 10^−7^	(1.0–3.0) × 10^−7^	Markedly weakened galvanic effect; dense stable passive film; uniform pitting corrosion	Micro-cutting + fatigue wear	[10,33,59,63,64]

## Data Availability

No new data were created or analyzed in this study. Data sharing is not applicable to this article.

## Abbreviations

The following abbreviations are used in this manuscript:

LAM	Laser additive manufacturing
LPBF	Laser powder bed fusion
DED	Directed energy deposition
C	Carbon
Nb	Niobium
N	Nitrogen
B	Boron
Cr	Chromium
Si	Silicon
O	Oxygen
Mn	Manganese
Mo	Molybdenum

## References

[1] Sun L., Liu Y., Li J., Chen K., Gao Y., Yang G. (2022). Morphology and microstructure of Fe–Cr–W–B alloy powders prepared by argon gas atomization. Vacuum.

[2] Holovenko Y., Antonov M., Kollo L., Hussainova I. (2018). Friction studies of metal surfaces with various 3D printed patterns tested in dry sliding conditions. Proc. Inst. Mech. Eng..

[3] Zhang C.L., Li S.H., Lin Y.H., Jiang J., Fu H. (2020). Effect of boron on microstructure evolution and properties of wear-resistant cast Fe-Si-Mn-Cr-B alloy. J. Mater. Res. Technol..

[4] Xu G.P., Wang K., Li H.N., Ju J., Dong X., Jiang H., Wang Q., Ding W. (2021). In situ nanoparticle-induced anti-oxidation mechanisms: Application to Fe-Cr-B alloys. Corros. Sci..

[5] Zhang J.J., Liu J.C., Liao H.M., Zeng M., Ma S. (2019). A review on relationship between morphology of boride of Fe-B alloys and the wear corrosion resistant properties and mechanisms. J. Mater. Res. Technol..

[6] Ling Z., Fu Z., Yang X., Lu T., Chen W. (2022). A new Fe-Cr-Mo-B-Al steel with outstanding tribo-corrosion resistance in liquid aluminium. Corros. Sci..

[7] Koga G.Y., Ferreira T., Guo Y., Coimbrão D.D., Jorge A.M., Kiminami C.S., Bolfarini C., Botta W.J. (2021). Challenges in optimizing the resistance to corrosion and wear of amorphous Fe-Cr-Nb-B alloy containing crystalline phases. J. Non Cryst. Solids.

[8] Zhang X., Liu B., Wang J., Zhou T. (2025). Effect of Bi on microstructure evolution of the Cr-Al-B MAB phase formed during hot-dip aluminizing and subsequent thermal diffusion treatment of Fe-Cr-B cast steel. Intermetallics.

[9] Ju J., Yang C., Ma S., Kang M., Wang K., Li J., Fu H., Wang J. (2020). Effect of temperature on oxidation resistance and isothermal oxidation mechanism of novel wear-resistant Fe-Cr-B-Al-C-Mn-Si alloy. Corros. Sci..

[10] Ling Z., Yang W., Wang X., Zhang Y., Jiang J., Chen X., Liu W., Geng Z., Peng Y. (2025). Enhanced corrosion resistance of laser-remelted Fe-Cr-B–Mo alloy through interfacial periodic layered structures in liquid aluminum. J. Mater. Res. Technol..

[11] Ling Z., Yang W., Wang X., Zhang X., Jiang J., Ni Z., Peng J., Yuan Z., Shi J., Chen W. (2024). Enhancing mechanical properties and corrosion resistance of Fe-Cr-B–Mo alloy via the ‘Divide and Conquer’ strategy for Ti regulation. J. Mater. Res. Technol..

[12] Wei L.N., Guo L.G., Guo D.G., Ten J.S.J., Yeong W.Y. (2024). Progress and Opportunities for Machine Learning in Materials and Processes of Additive Manufacturing. Adv. Mater..

[13] Ham G.S., Cho Y.H., Park S.Y., Kim C.P., Ko W.-S., Lee K.-A. (2023). Fabrication, microstructure, and wear properties of novel Fe-Cr-B–Nb–Mo metamorphic alloy coatings manufactured by the HVOF thermal spray process. Intermetallics.

[14] Sorour A.A. (2024). Fe-Cr-B-based wear-resistant alloys—A review on microstructure and tribological properties. J. Mater. Res. Technol..

[15] Ju J., Kang M., Zhou Y., Yang C., Wang K., Li J., Wang R., Fu H., Wang J. (2020). First-principles investigations of the stability, electronic structures, mechanical properties and thermodynamic properties of Fe_x_Al_y_C_z_ compounds in Fe-Cr-B-Al-C alloy. J. Phys. Chem. Solids.

[16] Zhang X., Li X., Ji K., Luo H., Chen Z. (2023). Formation mechanism of periodic layered structure between Fe–Cr–Si–B cast steel with high B content and molten Al. Mater. Des..

[17] Wang Y., Xu L., Zhao L., Han Y., Hao K., Ren W. (2023). Friction and tribocorrosion behavior of Fe-Cr-B alloys manufactured by laser directed energy deposition. Tribol. Int..

[18] Zhang X., Liu B., Ling Z. (2025). Growth of segmented Bi and Sn whisker on Cr-Al-B MAB phase formed during hot-dip aluminizing of Fe-Cr-B cast steel. Mater. Charact..

[19] Sun P., Liang J., Ma D., Tang C., Wang D., Lai T., Zhang H. (2025). Microstructural evolution and corrosion behavior of Fe-Cr-B alloy coatings: A comparative study between extreme high-speed and traditional laser cladding. Surf. Coat. Technol..

[20] Zhang C., Li X.M., Liu S.Q., Liu H., Yu L.-J., Liu L. (2019). 3D printing of Zr-based bulk metallic glasses and components for potential biomedical applications. J. Alloys Compd..

[21] Amiruddin H., Abdollah M.F.B., Norashid N.A. (2019). Comparative study of the tribological behaviour of 3D-printed and moulded ABS under lubricated condition. Mater. Res. Express.

[22] Joo Y.A., Yoon T.S., Park S.H., Lee K.-A. (2018). Microstructure and compression_properties of Fe-Cr-B alloy manufactured using laser metal deposition. Arch. Metall. Mater..

[23] Jian Y., Huang Z., Liu X., Sun J., Xing J. (2020). Microstructure, mechanical properties and toughening mechanism of directional Fe_2_B crystal in Fe-B alloy with trace Cr addition. J. Mater. Sci. Technol..

[24] Ni J., Ling H., Zhang S., Wang Z., Peng Z., Benyshek C., Zan R., Miri A., Li Z., Zhang X. (2019). Three-dimensional printing of metals for biomedical applications. Mater. Today Bio.

[25] Jian Y., Peng W., Ning H., Huang Z., Nie H., Hong J. (2024). Microstructure evolution and mechanical properties of Fe-Cr-B alloys with varying Mo additions. J. Mater. Res. Technol..

[26] Quan T., Zou B., Zhu J., Sun H., Ma X., Wang X. (2026). Dual-Laser SLA-3D printing system with partitioned scanning strategy for high-efficiency alumina ceramic fabrication. Opt. Laser Technol..

[27] Kelly B.E., Bhattacharya I., Heidari H., Shusteff M., Spadaccini C.M., Taylor H.K. (2019). Volumetric additive manufacturing via tomographic reconstruction. Science.

[28] Gao Y., Zhou M.Z. (2018). Superior Mechanical Behavior and Fretting Wear Resistance of 3D-Printed Inconel 625 Superalloy. Appl. Sci..

[29] Xia M.J., Gu D.D., Yu G.Q., Dai D., Chen H., Shi Q. (2016). Selective laser melting 3D printing of Ni-based superalloy: Understanding thermodynamic mechanisms. Sci. Bull..

[30] Dong K., Lv W., Sun Y., Wang P., Tian H., Zhou F., Du Z., Du C. (2025). Research on the dynamic mechanical properties of selective laser melting TC4 titanium alloy and the construction of J-C model. Mater. Today Commun..

[31] Shen W., Wang G., Wang S., Zhang Y., Kang J., Xiao Z., Fu X. (2025). Designing and vat photopolymerization 3D printing of glass ceramic/zirconia composites functionally gradient ceramics for dental restorations. Ceram. Int..

[32] Sun J., Wang Y., Zou J., Wade-Zhu J., Guo C., Shi Y., Bai J. (2024). Ceramic/metal composites fabricated via 3D printing and ultrasonic-assisted infiltration for high specific strength and energy absorption. J. Alloys Compd..

[33] Xiong F., Zou G., Qi Y., Sun B., Zhang B., Zhu S., Liang T., Zeng Q., Zhang X., Wang Q. (2026). Data-driven high-throughput screening of Fe-Cr-Mo-C-B amorphous alloy with excellent corrosion and wear resistance. Corros. Sci..

[34] Zhang F., Zhou S., You H., Zhang G., Yang J., Shi Y. (2025). 3D printing of ceramic matrix composites: Strengthening and toughening strategies. Compos. B Eng..

[35] Zhou Y., Chong X., Lin Y., Wang G., Jiang Y. (2024). Composition-dependent brittleness and ductility of the multicomponent phase (Fe, Cr)_23_(C, B)_6_ in Fe-Cr-B–C alloys: First-principles calculations and modeling. Vacuum.

[36] Mishra D.K., Giri J., Sathish T., Kanan M., Prajapati D. (2025). Influence of 3D printing LAM process parameters on mechanical properties of PLA based ceramic composite parts. Results Eng..

[37] Kaiser P., Malczyk P., Kerber F., Yaroshevskyi S., Weigelt C., Neumann M., Clague L., Hubálková J., Lohse U., Aneziris C.G. (2025). Fused filament fabrication based additive manufacturing of ceramic and metal-ceramic refractory parts for demanding thermal and chemical working conditions. Ceram. Int..

[38] Zhang Z., Feng Y., Li W., Liu X., Zhang X., Huang Y., Zhang K., Wan C. (2025). Stiff and ductile 3D-architectured metal/ceramic composites. Compos. A Appl. Sci. Manuf..

[39] Jian Y.X., Xing J.D., Huang Z.F., Wu T. (2019). Quantitative characterization of the wear interactions between the boride and metallic matrix in Fe-3.0 wt% B duplex alloy. Wear.

[40] Zhuang M.H., Li M.Q., Wang J., Ma Z., Zhou Z.-Y. (2017). Effect of Ti Addition on Phase Constitution and Wear Resistance of Fe-B-C Hypereutectic Overlays Produced Using Powder-Wire Composite Arc Welding. Mater. Trans..

[41] Manna I., Majumdar J.D., Chandra B.R., Nayak S., Dahotre N.B. (2006). Laser surface cladding of Fe-B-C, Fe-B-Si and Fe-BC-Si-Al-C on plain carbon steel. Surf. Coat. Technol..

[42] Ma X., Zhang W., Xiang Q., Ren Y., Yu B., Wang J., Qiu K. (2025). The microstructure and mechanical properties of three Fe-Cr-Mo-B bulk amorphous alloy composites. Mater. Lett..

[43] Dong Q., Song P., Tan J., Qin X., Li C., Gao P., Feng Z., Calin M., Eckert J. (2020). Non-isothermal crystallization kinetics of a Fe–Cr–Mo–B–C amorphous powder. J. Alloys Compd..

[44] Svoboda R., Chovanec J., Slang S., Beneš L., Konrád P. (2021). Single-curve multivariate kinetic analysis: Application to the crystallization of commercial Fe-Si-Cr-B amorphous alloys. J. Alloys Compd..

[45] Jin J., Sun J.S., Wang G.L. (2019). Effect of Mo content on microstructure and Wear resistance of Mo-Fe-B claddings. Int. J. Refract. Met. Hard Mater..

[46] Tian Y., Fu H.G. (2018). Development of Fe-Cr-B wear-resistant Alloys. China Foundry Mach. Technol..

[47] Zhang X., Liu B., Hu Q., Wang J. (2024). Interaction of the periodic layered structure formed on the hot-dip aluminized Fe-Cr-B cast steel with molten MoCl_5_ salt. Mater. Today Commun..

[48] Zhang X., Fu R., Liu B., Ling Z. (2025). Growth of various in whiskers on Cr-Al-B MAB phases formed during hot-dip aluminizing and subsequent thermal diffusion treatment of Fe-Cr-B cast steel. Intermetallics.

[49] Zhang X., Hu Q., Li X., Liu B., Ji K., Zheng Z., Luo H. (2024). On the effect of thermal diffusion treatment on the hot-dipped Al-Si alloy coating on Fe-Cr-B cast steel. Constr. Build. Mater..

[50] Carluccioac D., Berminghamac M., Kent D., Demir A.G., Previtali B., Dargusch M.S. (2019). Comparative Study of Pure Iron Manufactured by Selective Laser Melting, Laser Metal Deposition, and Casting Processes. Adv. Eng. Mater..

[51] Zhang G., Xu L.R. (2023). Simplified criterion to predict the lower bounds of the anisotropic ultimate strengths of 3D printing metals. Eng. Fail. Anal..

[52] Liu J., Zhang K., Bermingham M.J., Fraser H.L., Hodgson P., Heilmaier M., Boretti A., Zhu Y., Huang A. (2026). Fatigue and damage tolerance performance of additively-manufactured titanium alloys for structural application: A comprehensive review. Mater. Sci. Eng. R Rep..

[53] Liu Y., Su J., Li Y., Han R., Wong R.C.W., Hui J.H.P., Sing S.L. (2025). In-situ alloying modulation in additive manufacturing of titanium-tantalum alloy: From melt pool modelling to process development. Mater. Sci. Eng. R Rep..

[54] Röttger A., Lentz J., Theisen W. (2015). Boron-alloyed Fe–Cr–C–B tool steels—Thermodynamic calculations and experimental validation. Mater. Des..

[55] Xu G., Wang Q., Chen R., Li A., Chen D., Fu H. (2025). Columnar to equiaxed transition in additively manufactured titanium alloys: A comprehensive review of mechanisms and grain control strategies. J. Alloys Compd..

[56] Wang Z.W., Liu F., Gao S.B., Li Q., Chen K. (2026). Turn Nonweldable Ni-Superalloys Printable and Microstructurally Controllable. Adv. Mater..

[57] Gao J., Li T., Yan Z., Liu S., Zhao Y., Tong W. (2022). Research on the interface and properties of spherical ZTA particles reinforced Fe-Cr-B matrix composite. J. Mater. Res. Technol..

[58] Jonathan L., Arne R., Werner T. (2016). Mechanism of the Fe_3_(B, C) and Fe_23_(C, B)_6_ solid-state transformation in the hypoeutectic region of the Fe-C-B system. Acta Mater..

[59] Li G., Dong H., Zhang C., Li Y., Wang Y. (2025). Achieving wear resistance enhancement of Fe-Cr-B-C alloy coating by laser cladding and remelting processes. Opt. Laser Technol..

[60] Liu G.L., Liu S.C., Wang Q.H., Wang Y.-F., Xue W.-C., Guo S., Liu H.-X., Huang S., Lu J.-Z., Zhou J.-Z. (2025). Improving cavitation erosion resistance of laser-clad Fe–Mn–Si–Cr–Ni shape memory alloy coatings through B alloying. J. Mater. Res. Technol..

[61] Wang Y., Xu L., Zhao L., Han Y., Hao K., Ren W. (2024). Comparing electrochemical pitting behavior and passive films of Fe-Cr-B alloy coatings manufactured via high deposition rate and conventional laser directed energy deposition. Surf. Coat. Technol..

[62] Hou X.C., Du D., Wang K.M., Hong Y., Chang B. (2018). Microstructure and Wear Resistance of Fe-Cr-Mo-Co-C-B Amorphous Composite Coatings Synthesized by Laser Cladding. Metals.

[63] Zhang Q., Zhang P., Yan H., Yu Z., Wu D., Shi H., Li S., Tian Y. (2020). Magnetic-field-assisted laser cladding in the preparation of a crack-free Fe-Cr-Mo-C-Y-B amorphous coating on steel. Philos. Mag. Lett..

[64] Gou J., Wang Y., Zhang Y., Wang C., Wang G. (2021). Dry sliding wear behavior of Fe–Cr–C–B hardfacing alloy modified with nano-CeO_2_ and its mechanisms of modification. Wear.

[65] Ren J., Wu M., Li C.Y., Guan S., Dong J., Forien J.-B., Li T., Shanks K.S., Yu D., Chen Y. (2023). Deformation mechanisms in an additively manufactured dual-phase eutectic high-entropy alloy. Acta Mater..

[66] Pope A.D., Chen W., Chen H.M., Cao P., Yeghishyan A., Zhukovskyi M., Manukyan K., Vohra Y.K. (2024). High-pressure phase transition in 3D printed nanolamellar high-entropy alloy by imaging and simulation insights. Sci. Rep..

[67] Wei M.W., Chen S.Y., Liang J., Liu C. (2017). Effect of atomization pressure on the breakup of TA15 titanium alloy powder prepared by EIGA method for laser 3D printing. Vacuum.

[68] Agbedor S.O., Wu H., Ren Y., Liang L., Yang D., Liu B., Liu Y., Baker I. (2024). A two-decade odyssey in fusion-based additive manufacturing of titanium alloys and composites. Appl. Mater. Today.

[69] Zhao Y.H., Wang Z.G., Zhao J.B., Shi F. (2018). Microstructure and Properties of Laser Additive Manufactured Fe-Cr-Ni-B Steel by Divided-area Process. Rare Met. Mater. Eng..

[70] Chen M., Zhou H.R., Liu X.Y., Feng Z., Xiao X., Weng L., Yang Y., Jiang Y. (2025). Morphology and Wear Resistance of Laser-Clad Fe-Cr-Nb-C Alloy Coatings. Coatings.

[71] Gao J., Yan Z.L., Liu S., Zhao Y., Li T., Tong W. (2023). Microstructure and mechanical properties of a Mo alloyed Fe-Cr-B alloy. Vacuum.

[72] Xu G.P., Wang K., Dong X.P., Yang L., Jiang H., Wang Q., Ding W. (2020). Effects of Titanium Addition on the Microstructural and Mechanical Property Evolution of Fe-Cr-B Alloys. Metall. Mater. Trans. A.

[73] Su J.L., Chen L.Q., Van Petegem S., Jiang F., Li Q., Luan J., Sing S.L., Wang J., Tan C. (2025). Additive manufacturing metallurgy guided machine learning design of versatile alloys. Mater. Today.

[74] Wang Y.W., Xu L.Y., Zhao L., Han Y., Hao K., Ren W. (2024). Tribo-corrosion performance of Fe-Cr-B alloy coating manufactured by high deposition rate and conventional laser directed energy deposition. Tribol. Int..

[75] Zhang H., Zhang Y., Ye C., Rotella G., Umbrello D. (2025). The effects of laser assisted ultrasonic nanocrystal surface modification on 3D-printed Ti6Al4V alloy. CIRP J. Manuf. Sci. Technol..

[76] Song K., Wang Y.W., Liu M.Q., Ma C., Xu L., Han Y., Zhao L. (2026). The tribo-corrosion properties of Fe-18Cr-B alloy fabricated by laser directed energy deposition. Corros. Sci..

[77] Ma S.Q., Xing J.D., He Y.L., Fu H., Li Y., Liu G. (2016). Effect of orientation and lamellar spacing of Fe_2_B on interfaces and corrosion behavior of Fe-B alloy in hot-dip galvanization. Acta Mater..

[78] Wang J.J. (2016). Brief introduction of atomizing and milling technology in China. Powder Metall. Ind..

[79] Tang X., Li A.H., Li B. (2018). Preparation of spherical titanium and titanium alloy powder. Powder Metall. Ind..

[80] Franz H., Plöchl L., Schimansky F.P. (2008). Recent Advances of Titanium Alloy Powder Production by Ceramic-free Inert Gas Atomization. Titan. Org..

[81] Li A., Liu S.F., Wang B.J., Zhang Z.H., Shi M.J. (2018). Research progress on preparation of metal powder for 3D printing. J. Iron Steel Res..

[82] Nan Y. (2025). Application of Machine Learning Based on Big Data in Metal 3D Printing. Procedia Comput. Sci..

